# Assessing the Causal Relationship between Genetically Determined Inflammatory Cytokines and Parkinson's Disease Risk: A Bidirectional Two-Sample Mendelian Randomization Study

**DOI:** 10.1155/2024/9069870

**Published:** 2024-02-29

**Authors:** Hua Xue, Qian Luo, Jiajia Chen, Wenhui Fan

**Affiliations:** ^1^Department of Neurology, Sichuan Taikang Hospital, Chengdu, Sichuan 610213, China; ^2^Department of Dermatology, Jian Yang Hospital of Traditional Chinese Medicine, Chengdu, Sichuan, China; ^3^Sichuan University of Science and Engineering, Zigong, Sichuan, China

## Abstract

**Background:**

Observational studies have suggested an association between inflammatory cytokines and Parkinson's disease (PD). This Mendelian randomization (MR) was conducted to further assess the causal correlations between inflammatory cytokines and PD.

**Methods:**

Genetic instruments associated with inflammatory cytokines were extracted from a large summary genome-wide association studies (GWAS) involving 8,293 European participants. Summary-level statistics for PD were obtained from a large-sample GWAS containing 17 studies that involved European participants. Causalities of exposures and outcomes were explored mainly using inverse variance weighted (IVW) method.

**Results:**

The IVW method indicated that basic fibroblast growth factor (FGFBasic), interleukin-2 (IL-2), and macrophage migration inhibitory factor (MIF) may be suggestively associated with the risk of PD (OR: 0.71, 95%CI: 0.52–0.96, *P* = 0.027; OR: 1.18, 95%CI: 1.01–1.38, *P* = 0.041; and OR: 1.23, 95%CI: 1.04–1.46, *P* = 0.018). In the reverse direction, monokine induced by interferon gamma (MIG), beta nerve growth factor (bNGF), interleukin-17 (IL-17), and interferon gamma (IFNg) are suggested to be the consequences of PD.

**Conclusion:**

Our MR analysis indicated that suggestive associations between circulating levels of FGFBasic, IL-2, and MIF and PD risk. In addition, MIG, bNGF, IL-17, and IFNg are more likely to be involved in the development of downstream PD.

## 1. Introduction

Parkinson's disease (PD) is a common neurodegenerative disease that occurs in middle-aged and elderly individuals, with insidious onset and slow progression [1]. Its characteristic pathological changes are progressive degenerative reduction of nigrostriatal dopaminergic neurons and the formation of Lewy bodies, which leads to a reduction of dopamine transmitters in striatal regions [2]. The clinical manifestations of PD are primarily characterized by symptoms such as bradykinesia, resting tremor, myotonia, and postural balance disorders. These symptoms are often accompanied by a range of nonmotor symptoms, including olfactory disorders, cognitive disorders, mental disorders, constipation, and sleep disorders [3]. The diagnosis of PD primarily depends on a comprehensive medical history and a thorough neurological physical examination. Currently, there is no specific test available for diagnosing PD. The exact cause of PD remains incompletely understood, and there are no reliable clinical or testing methods to determine its cause. However, most scholars currently believe that PD is influenced by a combination of age factors, environmental factors, and genetic factors [4, 5]. According to epidemiological studies, the prevalence of PD among individuals aged 60 and above in European and American countries is approximately 1% [6]. Moreover, the prevalence of PD among individuals over 80 years old exceeds 4%. In 2016, the global number of PD patients was estimated to be around 6.1 million [7]. As the disease advances, both the motor and nonmotor symptoms of PD progressively worsen. This not only hampers the patient's daily activities but also imposes a significant burden on the patient's family and society.

Several studies have confirmed that the degenerative necrosis of midbrain nigrostriatal dopamine neurons is the primary pathological change in PD [8]. The immune-inflammatory response is closely associated with both central neurodegeneration and nigrostriatal–striatal damage, which may contribute to the onset and progression of PD, attracting significant attention [8]. Various factors, such as neuronal degeneration, microglia activation, infiltration of peripheral blood lymphocytes, and disruption of the blood–brain barrier (BBB) caused by the inflammatory response have been implicated as etiological factors in PD [9]. Neuronal degeneration, activation of microglia, invasion of peripheral blood lymphocytes, and damage to the BBB caused by inflammatory reactions have become the causes of PD [10]. Microglia activated upon external stimuli upregulate a variety of cellular inflammatory factors through the nuclear transcription factor pathway, and these inflammatory factors include tumor necrosis factor-*α* (TNF-*α*), interleukin-1*β* (IL-1*β*), IL-6, and others [11]. The expression of inflammatory factors is then involved in the necrosis and damage of dopaminergic neurons, and these inflammatory factors can add to the degeneration and loss of neurons. TNF-*α* can activate caspase-specific protease (Caspase) directly and contribute to neuronal necrosis and apoptosis through the apoptosis mechanism [12]. According to Muller and Beharka et al., IL-6 inflammatory cytokines have the potential to repair neurons and promote neuron regeneration in patients with PD [13, 14]. Furthermore, it has been suggested by some researchers that the decrease in the number of glial cells during the progression of the disease results in a significant reduction in peripheral blood IL-6. However, there is currently no definitive evidence to determine whether alterations in peripheral blood IL-6 levels have a detrimental or protective impact on neurons.

In the treatment of PD, there has been extensive discussion on reducing levels of inflammatory cytokines to inhibit the progression of PD [15]. However, there are limited observational studies that link specific circulating inflammatory cytokines to the risk of PD, and these studies have relatively small sample sizes [16]. Additionally, the results of these studies may be influenced by confounders, reverse causality, and other biases that were not measured. To address these potential limitations and strengthen the evidence for a potential causal role of circulating inflammatory cytokines in PD risk, Mendelian randomization (MR) can be implemented [17]. MR is a method that uses genetic variation as an instrumental variable (IV) to investigate causal associations between exposures and outcomes. Since genetic variation is randomly inherited, MR can be considered as a natural randomized controlled trial (RCT) [18]. In this study, we extracted valid genetic variants from pooled data of 41 inflammatory cytokines from published genome-wide association studies (GWAS) to examine their association with PD. We also explored the direction of causation by reversing the exposure and outcome.

## 2. Methods

### 2.1. Study Design

**Figure 1:**
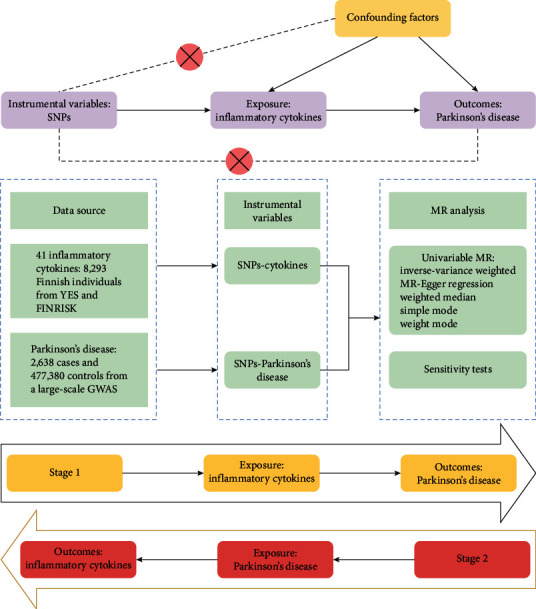
Overview of the assumptions of the Mendelian randomization (MR) design and the study design.

The bidirectional MR study flow for this study is shown in Figure 1. No additional ethical approval was required as we used pooled statistics from published studies. MR analysis was performed following three key assumptions, namely correlation, independence, and exclusion restrictions [19]. The selected genetic variants were highly correlated with risk factors (correlation) but not with any confounders in the outcome associations (independence), and they did not influence the outcome in any way other than the associated risk factors (exclusion restriction) [20]. In this bidirectional study, we utilized genetic variants associated with 41 systemic inflammatory cytokines and PD extracted from published GWAS. This study followed the Strengthening the Reporting of Observational Studies in Epidemiology Using Mendelian Randomization (STROBE-MR) reporting guidelines [21].

### 2.2. Data Sources

We extracted genetic variation from published large-scale GWAS meta-analyses of circulating concentrations of 41 inflammatory cytokines in 8,293 European participants from three independent cohorts: the Finnish Young People's Cardiovascular Risk Study (YFS), FINRISK1997, and FINRISK2002 [22]. Quantitative analyses of cytokines were performed from FINRISK 1997 ethylenediaminetetraacetic acid plasma, FINRISK 2002 heparin plasma, and serum from the YFS, and were measured using the Bio-Plex Pro Human Cytokine 27-plex Assay, the Bio-Plex 200 reader, and the Bio-Plex 6.0 software [22]. The mean age of participants in the YFS study was 37 years. The mean age of participants in the FINRISK investigation was 60 years. Genetic associations were meta-analyzed for the three cohorts. We collected summary data of PD from a large-sample GWAS containing 17 studies that involved only European participants (2,638 cases and 477,380 controls) [23]. Participants had an identical genetic background, and there was no overlap between exposure GWASs and outcome GWASs.

### 2.3. Selection of Genetic Instruments

To fulfill the three key assumptions of the MR analysis, we selected genetic variants as IVs that met the following criteria: (1) genetic variants must be closely associated with exposure. We used *P* < 5 × 10^−8^ as the genome-wide significance threshold to select single nucleotide polymorphisms (SNPs) that are strongly associated with PD and inflammatory cytokines. Since few SNPs were identified as IVs when using inflammatory cytokines as exposure, we selected SNPs with *P* < 5 × 10^−6^ as IVs for 41 inflammatory cytokines [19]; (2) genetic variants were assayed by linkage disequilibrium (LD) with the parameters set at 10,000 kb, *R*^2^ < 0.001, to identify SNPs in LD status, and these SNPs were isolated [20]; (3) when merging exposure data and outcome data, use the “harmonise” function and “action = 2” to remove palindromic sequences; (4) to exclude potential multiple effects, we searched for secondary phenotypes of each SNP in PhenoScanner V2, and SNPs corresponding to phenotypes unrelated to exposure were excluded, and we eliminated those that were missing from the results and had not been identified by R software to identify appropriate alternative SNPs, and the remaining SNPs were used for further analysis [24]; and (5) for the screened IVs, we assessed the strength of the IVs using the variance (*R*^2^) and the F statistic, and the correlation between the IVs and the exposure was considered to be sufficiently strong if *F* > 10 and the results of the MR analyses were protected from weak instrumental bias [25]. *F* = *R*^2^ (NK−1)/(K(1−*R*^2^)), where *R*^2^ refers to the cumulative explained variance of the selected SNPs during exposure, K is the number of SNPs finally analyzed, and N is the sample size of the selected GWAS [26].

### 2.4. Statistical Analyses

Five MR analysis methods were conducted in this study to assess the causal association between inflammatory cytokines and PD, including the inverse variance weighted (IVW) method, the weighted median (WM) method, the MR-Egger method, the simple model, and the weighted model. The IVW method is the main method of MR analysis and is considered to be the most effective method to evaluate the causal effect [27]. The premise of the IVW method is that all genetic variations are valid instrumental variables, but may not be established in practice [27]. Therefore, we also use other robust methods to give a consistent estimate of causal parameters without the need for all genetic variations to be valid IVs. The WM method is more tolerant of invalid IVs, allowing at least half of the IVs to be valid [26]. The MR-Egger method provides causal estimates even when all IVs are invalid [27].

To determine whether IVs have unbalanced pleiotropic effects that lead to bias, we performed the Mendelian randomization pleiotropy residual sum and outlier (MR-PRESSO) method and MR-Egger regression intercept. We calculated the intercept of the MR-Egger regression, and *P*  > 0.05 suggested the presence of horizontal pleiotropy [28]. MR-PRESSO is based on the IVW regression framework and detects horizontally ambiguous IVs as outliers in regression [29]. The MR-PRESSO method detects possible IV outliers through global testing and provides unbiased causal estimates by eliminating identified outliers. We used Cochran's Q statistic to quantify heterogeneity, and *P*  < 0.05 was considered significant heterogeneity [26, 30]. We also performed a leave-one-out sensitivity analysis, leaving out each SNP in turn to determine whether a specific variant drove the association between exposure and outcome, and applying an IVW approach to the remaining SNPs.

The results are reported as effect sizes (ESs) along with their corresponding 95% confidence intervals (CIs). All statistical analyses were conducted using two-sided tests. A *P*-value of less than 0.0012 (adjusted to 0.05/41 using the Bonferroni method) was considered statistically significant, while a *P*-value between 0.0012 and 0.05 was considered suggestive. The TwoSampleMR and MRPRESSO software packages in R version 4.2.2 were utilized for all analyses.

## 3. Results

### 3.1. Causal Effects of Different Inflammatory Cytokines on the Risk of Parkinson's Disease

**Figure 2:**
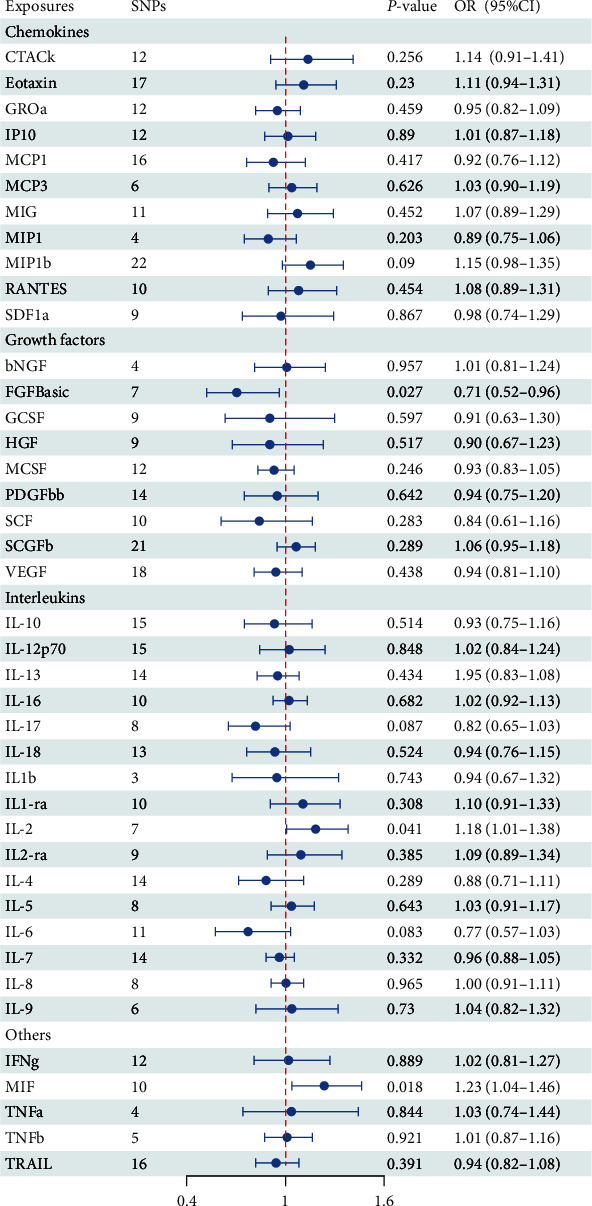
Causal correlations of 41 inflammatory cytokines on Parkinson's disease in inverse variance weighted method. bNGF, beta nerve growth factor; CTACK, cutaneous T cell-attracting chemokine; FGFBasic, basic fibroblast growth factor; GCSF, granulocyte colony-stimulating factor; GROa, growth-regulated oncogene-a; HGF, hepatocyte growth factor; IFNg, interferon gamma; IL, interleukin; IP, interferon gamma-induced protein 10; MCP1, monocyte chemotactic protein 1; MCP3, monocyte-specific chemokine 3; MCSF, macrophage colony-stimulating factor; MIF, macrophage migration inhibitory factor; MIG, monokine induced by interferon gamma; MIP1a, macrophage inflammatory protein-1a; MIP1b, macrophage inflammatory protein−1b; PDGFbb, platelet-derived growth factor BB; RANTES, regulated upon activation normal T cell expressed and secreted factor; SCF, stem cell factor; SCGFb, stem cell growth factor beta; SDF1a, stromal cellderived factor-1 alpha; SNPs, single-nucleotide polymorphisms; TNFa, tumor necrosis factor alpha; TNFb, tumor necrosis factor beta; TRAIL, TNF-related apoptosis-inducing ligand; VEGF, vascular endothelial growth factor; and OR, odd ratio.

In three independent population cohorts, all 41 inflammatory cytokines using the less stringent cutoff value of *P* < 5 × 10^−6^ had three or more SNPs with F statistics ranging from 20.83 to 132.61, suggesting that the weak instrumentation bias was not significant (Tables S1–S3).  Figure 2 shows the causal relationship between 41 systemic inflammatory cytokines and PD risk in the IVW method.

Regarding basic fibroblast growth factor (FGFBasic), we identified a suggestive association between circulating FGFBasic levels and PD risk in IVW analysis. Specifically, for one SD decrease of FGFBasic levels, the OR of PD risk was 0.71 (odd ratio (OR): 0.71, 95% CI: 0.52–0.96; *P* = 0.027, Figure S1). Further analysis showed a lack of evidence of heterogeneity among SNPs in the suggestive association of FGFBasic with PD risk as measured by Cochran's Q test (*P* = 0.258; Table S2). In addition, no potential pleiotropy was detected using the MR-Egger method (Table S2). For interleukin-2 (IL-2), we found by the IVW method that genetically determined higher IL-2 levels (one-SD increase) were suggestively associated with 18% higher odds for PD (OR: 1.18, 95%CI: 1.01–1.38, *P* = 0.041, Figure S2). Furthermore, we did not observe any significant heterogeneity as measured by Cochran's Q test (*P* = 0.117) and no evidence of potential pleiotropy measured by MR Egger method (*P* = 0.947). Regarding macrophage migration inhibitory factor (MIF), we identified a suggestive association between circulating MIF levels and PD risk in IVW analysis (OR: 1.23, 95%CI: 1.04–1.46, *P* = 0.018, Figure S3). The scatter plots MR analyses for FGFBasic, IL-2, and MIF on PD are exhibited in FIgures S1–S3. Meanwhile, the *P*-values for the intercepts from Egger regression did not demonstrate any pleiotropy (*P* = 0.987) and no evidence of heterogeneity measured by Cochran's Q test (*P* = 0.789).

Apart from FGFBasic, IL-2, and MIF, the other 38 inflammatory cytokines were not shown to be associated with PD risk in the main IVW analysis and four supplementary analyses (Table S1). For each cytokine, no marked heterogeneity was found between related SNPs, except for granulocyte colony-stimulating factor (GCSF) and IL-18 (all *P*  < 0.05). Meanwhile, the *P*-values for the intercepts from Egger regression did not demonstrate any pleiotropy.

### 3.2. Causal Impact of Parkinson's Disease on Different Inflammatory Cytokines

**Figure 3:**
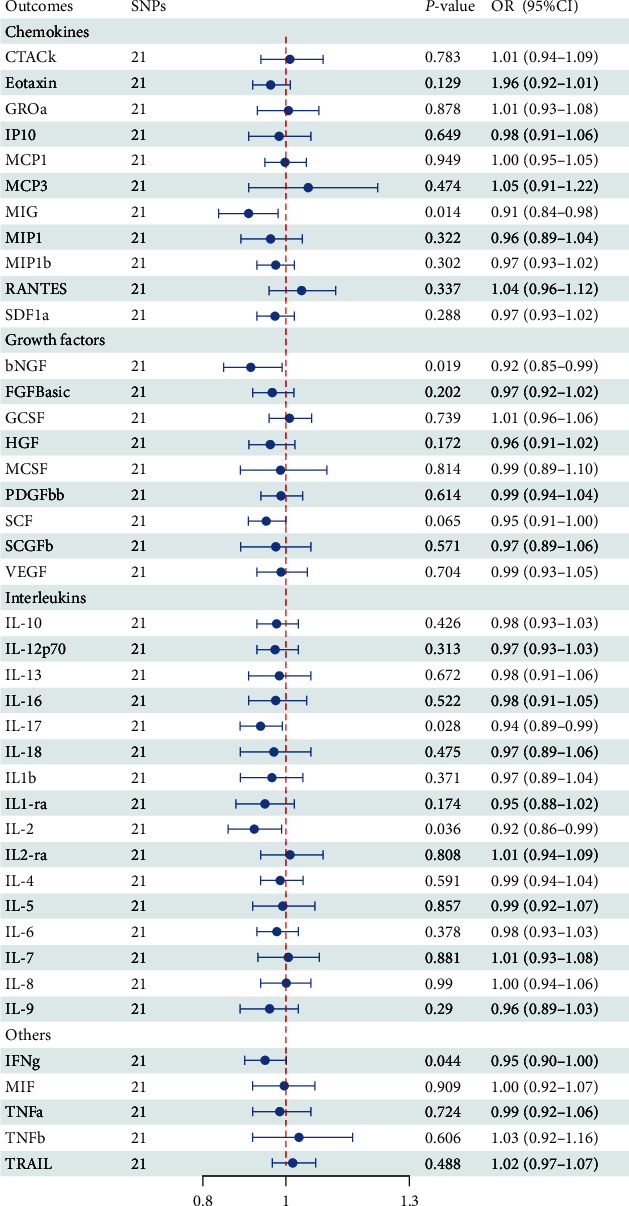
Causal correlations of Parkinson's disease on 41 inflammatory cytokines in inverse variance weighted method. bNGF, beta nerve growth factor; CTACK, cutaneous T cell-attracting chemokine; FGFBasic, basic fibroblast growth factor; GCSF, granulocyte colony-stimulating factor; GROa, growth-regulated oncogene-a; HGF, hepatocyte growth factor; IFNg, interferon gamma; IL, interleukin; IP, interferon gamma-induced protein 10; MCP1, monocyte chemotactic protein 1; MCP3, monocyte-specific chemokine 3; MCSF, macrophage colony-stimulating factor; MIF, macrophage migration inhibitory factor; MIG, monokine induced by interferon gamma; MIP1a, macrophage inflammatory protein-1a; MIP1b, macrophage inflammatory protein-1b; PDGFbb, platelet-derived growth factor BB; RANTES, regulated upon activation normal T cell expressed and secreted factor; SCF, stem cell factor; SCGFb, stem cell growth factor beta; SDF1a, stromal cellderived factor−1 alpha; SNPs, single-nucleotide polymorphisms; TNFa, tumor necrosis factor alpha; TNFb, tumor necrosis factor beta; TRAIL, TNF-related apoptosis-inducing ligand; VEGF, vascular endothelial growth factor; OR, odd ratio.

Overall, 21 SNPs significantly associated with PD were identified at the genome-wide significant level (*P* < 5 × 10^−8^) and LD based on *R*^2^ < 0.001. F statistics ranged from 30.02 to 181.49, suggesting that the results of MR analyses are rarely affected by weak instrumental variables (Tables S4–S6). Detailed information on the reverse IVW analysis is shown in Figure 3 and Table S4.

In the reverse MR analysis, we did not detect heterogeneity and horizontal pleiotropy, so the IVW method was used as the primary analysis of PD with inflammatory factors (Tables S4–S6). The findings of the IVW method demonstrated that PD was suggestively correlated with an decreased level of monokine induced by interferon gamma (MIG; OR: 0.91, 95%CI: 0.84–0.98, *P* = 0.014), beta nerve growth factor (bNGF; OR: 0.92, 95%CI: 0.85–0.99, *P* = 0.019), interleukin-17 (IL-17; OR: 0.94, 95%CI: 0.89–0.99, *P* = 0.028), IL-2 (OR: 0.92, 95%CI: 0.86–0.99, *P* = 0.036), and interferon gamma (IFNg; OR: 0.95, 95%CI: 0.90–1.00, *P* = 0.044). The scatter plots MR analyses for PDF on MIG, bNGF, IL-17, and IFNg are exhibited in Figure S4–S8. Apart from MIG, bNGF, IL-17, IL-2, and IFNg, the other 36 inflammatory cytokines were not shown to be associated with PD in the reverse IVW analysis and four supplementary analyses (Table S4).

## 4. Discussion

In this study, we conducted a two-sample MR analysis using the largest publicly available GWAS data set to explore potential causal relationships between 41 inflammatory cytokines and PD. We examined 41 inflammatory cytokines, including growth factors, interleukins, and chemokines, as exposure variables, with PD as the outcome. Our findings suggest that FGFBasic, IL-2, and MIF may be involved in the development of PD as upstream factors. Additionally, when PD is considered as an exposure variable in MR, it may lead to decreased levels of MIG, bNGF, IL-17, IL-2, and IFNg through pathogenic pathways. These results indicate that several biomarkers could potentially initiate PD, while other inflammatory regulators are more likely to be downstream factors in the progression of the disease.

Previous studies have demonstrated a strong link between PD and inflammatory biomarkers [31, 32]. For example, a Meta-analysis study involving 25 studies based on 25 inflammatory biomarkers containing a case group of 1,547 patients and a control group of 1,107 patients found that patients with PD had elevated levels of inflammatory cytokines, providing clinical evidence in support of the inflammatory response accompanying the disease. Cytokines significantly increased in PD patients compared to healthy individuals included IL-6, TNF, IL-1*β*, IL-2, IL-10, and C-reactive protein (CRT), as well as the chemokines, which is associated with inflammatory cell infiltration [33]. Moreover, a study was conducted to evaluate the plasma levels of inflammatory vesicle-associated proteins and the downstream inflammatory cytokine IL-18 in 32 patients with PD and compared them with age-matched unaffected controls [34]. The study findings suggest that levels of Caspase-1 and IL-18 proteins were significantly higher in patients with PD compared to controls. The researchers assessed the reliability of each protein as a biomarker of inflammation in PD by plotting their subject operating characteristic (ROC) curves. Caspase-1 showed an AUC value of 0.96, a specificity of 85%, and a sensitivity of 96.88%. The inflammatory cytokine IL-18 had an AUC value of 0.85, a specificity of 75%, and a sensitivity of 90.63% [34]. Furthermore, a multiple linear regression analysis using a stepwise approach was performed by the researchers, demonstrating that inflammatory vesicle proteins are reliable biomarkers of inflammation in PD. Additionally, it was found that inflammatory vesicle proteins significantly contribute to IL-18 levels in PD [34].

Neuroinflammation is a common pathologic feature of several central nervous system diseases [35]. It has been reported that neuroinflammation plays the role of a double-edged sword in the nervous system [35]. A moderate inflammatory response can remove necrotic cells and toxic proteins and maintain the stability of the blood–brain barrier, which is conducive to the recovery of the disease, but an excessive inflammatory response can cause a large amount of inflammatory cytokines to be released, which can damage the blood–brain barrier, mitochondrial function, and cellular energy metabolism, and aggravate the damage of brain tissue [36]. In our forward MR analysis, IVW results suggest that FGFBasic, IL-2, and MIF may be involved in the development of PD as upstream factors. FGFBasic is a multifunctional peptide growth factor that activates intracellular signaling cascades by binding to tyrosine kinase fibroblast growth factor receptors (FGFRs) [37]. FGFs are widely present in various organisms and have crucial roles in cellular processes through paracrine, autocrine, or endocrine functions. They are involved in embryonic development, angiogenesis, tissue homeostasis, wound repair, and cancer genesis and development [38]. During embryonic development, FGF regulates cell proliferation, differentiation, and migration, contributing to morphogenesis. In adults, FGF serves as a homeostatic factor, regulating tissue repair, wound healing, nervous system control, and tumor angiogenesis [38]. IL-2, a growth factor related to T cells, has the ability to enhance the killing activity of NK cells and stimulate the production of immunoglobulins by B cells [39]. It also plays a role in the development of regulatory T cells (Tregs), which contribute to peripheral T cell immune tolerance and regulate the proliferation and differentiation of activated T cells [40]. Tregs, as important immune negative regulatory cells, play a crucial role in various neurological diseases. Loss of Tregs exacerbates the inflammatory response in mouse models of multiple sclerosis (MS), stroke, or traumatic brain injury, leading to worsened disease progression [41]. IL-2 is vital for the survival and stability of Tregs, and low-dose IL-2 has shown promising outcomes in different autoimmune disease models. Yshii et al. [41] discovered the regulatory effect of IL-2 on Treg cells in the brain and proposed an astrocyte-based gene delivery system capable of crossing the blood–brain barrier and enhancing the immune response. They observed IL-2 secretion by astrocytes and its protective effects on the nervous system in mouse models of traumatic brain injury, stroke, and MS [41]. Parthanatos-associated apoptosis-inducing factor nuclease (PAAN), also known as macrophage migration inhibitory factor (MIF), is a member of the PD-D/E(X)K nuclease family [42]. It serves as the final executioner in parthanatos [43]. In a study by Park et al. [44], it was demonstrated that pathologic *α*-synuclein (*α*-syn) triggers neurological degeneration through the activity of PAAN/MIF nuclease. Deletion of the PAAN/MIF gene and a mutant lacking nuclease activity effectively prevented dopaminergic neuronal deficits and behavioral defects in the *α*-syn preformed fiber (PFF) mouse model [44]. Consistent with the findings of many previous observational studies, altered levels of MIF and IL-2 were associated with the risk of PD. This may be attributed to the potential role of an active inflammatory response in neuronal degeneration.

In reverse MR analysis, we found that PD affects the levels of MIG, bNGF, IL-17, IL-2, and IFNg through pathological pathways. bNGF, a member of the neurotrophic factors family, consists of *β* subunits. It acts as a regulator for nerve cell growth, with dual functions of neurotrophic support and promoting neurite growth. bNGF plays a crucial role in regulating the development, differentiation, growth, regeneration, and functional characteristics of both the central and peripheral nervous systems [45]. IL-17 is closely associated with chronic inflammatory diseases such as MS and arthritis [46]. It is a highly conserved component of the vertebrate immune system and plays a crucial role in regulating infections and autoimmune diseases [46]. Regen et al. [46] demonstrated that mice lacking IL-17 are less susceptible to experimental autoimmune encephalomyelitis (EAE). However, when the bacterial flora is restored, their susceptibility to EAE is also restored. Moreover, restoring the expression of IL-17 in the intestinal epithelium can also reinstate the susceptibility of IL-17-deficient mice to EAE. These findings suggest that IL-17 indirectly modulates autoimmune diseases of the central nervous system through the influence of intestinal flora [47]. Inconsistent with the results of previous studies, our findings suggest that PD leads to reduced levels of inflammatory factors such as MIG, bNGF, IL-17, IL-2, and IFNg through pathological pathways, which may be attributed to the fact that the levels of inflammatory markers are influenced by the course, extent, and duration of PD.

## 5. Strengths

To our knowledge, no MR studies have been reported on the causal effect of inflammatory markers on PD or vice versa. Our study utilized multiple IVs from GWAS of inflammatory markers and PD to increase the statistical efficacy of detecting causality, providing a more precise assessment of effect size. According to our MR analysis, there is a causal relationship between certain inflammatory factors and PD (FGFBasic, IL-2, and MIF, etc.). Therefore, it is crucial to identify and predict PD at an early stage. We encourage researchers to focus on studying PD and inflammatory factors, actively search for risk factors associated with PD, explore predictive markers for the development and progression of PD, and offer early intervention and treatment.

## 6. Conclusion

In conclusion, this MR analysis shows suggestive associations between circulating levels of FGFBasic, IL-2, and MIF and PD risk. In addition, MIG, bNGF, IL-17, and IFNg are more likely to be involved in the development of downstream PD. Our findings bring new insights into the pathogenesis of PD.

## Data Availability

The original contributions presented in the study are included in the article/Supplementary Materials. Further inquiries can be directed to the corresponding authors.

## Additional Points

*Limitations*. Our study has several limitations. Firstly, the second and third hypotheses could not be accurately tested due to the constraints of MR analysis, potentially introducing bias. Secondly, our survey data came from two large-scale global genomic studies, and due to the lack of specific demographic information and clinical records, it was not possible to analyze subgroups of Parkinson's patients, such as early-onset PD, male PD, female PD, etc. Thirdly, our data only included assessments of bioinflammatory marker concentrations in peripheral blood, rather than cerebrospinal fluid samples. Analyzing the inflammatory components in cerebrospinal fluid could provide a clearer understanding of the neuroinflammatory process underlying PD. Fourthly, although we have learned that the functional impact of PD may be altered by a series of interactions between cytokines, there is a lack of a series of experiments (e.g., cytokine profiling by enzyme linked immunosorbent assay (ELISA) or multiplex assays) to understand complex interaction of cytokines in PD pathology. We hope that future researchers will undertake a series of experiments on PD to gain a better understanding of the complex cytokine interactions involved in its pathology. Lastly, the findings may not be universally applicable, and caution is needed when extrapolating the conclusions to other ethnicities, as the genetic and environmental factors influencing PD may vary across populations.

## Supplementary Materials

Supplementary Materials Table S1: MR estimates of 41 inflammatory cytokines on Parkinson's disease. Table S2: heterogeneity and horizontal pleiotropy tests of forty-one inflammatory cytokines on Parkinson's disease. Table S3: SNPs information of forty-one inflammation cytokines with Parkinson's disease. Table S4: MR estimates of PD on forty-one inflammatory cytokines. Table S5: heterogeneity and horizontal pleiotropy tests of PD on forty-one inflammatory cytokines. Table S6: detail information of instrumental variables of PD. Figure S1: scatter plots of Mendelian randomization analyses for FGFBasic on PD. Figure S2: scatter plots of Mendelian randomization analyses for IL-2 on PD. Figure S3: scatter plots of Mendelian randomization analyses for MIF on PD. Figure S4: scatter plots of Mendelian randomization analyses for PD on MIG. Figure S5: scatter plots of Mendelian randomization analyses for PD on bNGF. Figure S6: scatter plots of Mendelian randomization analyses for PD on IL-17. Figure S7: scatter plots of Mendelian randomization analyses for PD on IL-2. Figure S8: scatter plots of Mendelian randomization analyses for PD on IFNg.
