# Supplementary material for: Assessing the Causal Relationship between Genetically Determined Inflammatory Cytokines and Parkinson's Disease Risk: A Bidirectional Two-Sample Mendelian Randomization Study
Source: J Immunol Res. 2024 Feb 29;2024:9069870. doi: 10.1155/2024/9069870 (PMC10919978; doi:10.1155/2024/9069870)
Supplement: Supplementary Materials — Table S1: MR estimates of 41 inflammatory cytokines on Parkinson's disease. Table S2: heterogeneity and horizontal pleiotropy tests of forty-one inflammatory cytokines on Parkinson's disease. Table S3: SNPs information of forty-one inflammation cytokines with Parkinson's disease. Table S4: MR estimates of PD on forty-one inflammatory cytokines. Table S5: heterogeneity and horizontal pleiotropy tests of PD on forty-one inflammatory cytokines. Table S6: detail information of instrumental variables of PD. Figure S1: scatter plots of Mendelian randomization analyses for FGFBasic on PD. Figure S2: scatter plots of Mendelian randomization analyses for IL-2 on PD. Figure S3: scatter plots of Mendelian randomization analyses for MIF on PD. Figure S4: scatter plots of Mendelian randomization analyses for PD on MIG. Figure S5: scatter plots of Mendelian randomization analyses for PD on bNGF. Figure S6: scatter plots of Mendelian randomization analyses for PD on IL-17. Figure S7: scatter plots of Mendelian randomization analyses for PD on IL-2. Figure S8: scatter plots of Mendelian randomization analyses for PD on IFNg. [file 9069870.f1.docx]

Supplementary Description

**Causal role of inflammatory cytokines in Parkinson’ s disease: a bidirectional two-sample Mendelian randomization study**

**Table S1.** MR estimates of forty-one inflammatory cytokines on Parkinson’s disease

|  | | **Inverse variance weighted** | | | | **MR-Egger** | | | **Weighted Median** | | | **Simple Mode** | | | **Weighted Mode** | | |
| --- | --- | --- | --- | --- | --- | --- | --- | --- | --- | --- | --- | --- | --- | --- | --- | --- | --- |
| **Category** | **Exposures** | **SNPs** | **OR** | **95% CI** | **pval** | **OR** | **95% CI** | **pval** | **OR** | **95% CI** | **pval** | **OR** | **95% CI** | **pval** | **OR** | **95% CI** | **pval** |
| **Chemokines** |  |  |  |  |  |  |  |  |  |  |  |  |  |  |  |  |  |
|  | CTACK | 12 | 1.135 | (0.91-1.41) | 0.256 | 0.858 | (0.57-1.29) | 0.481 | 1.024 | (0.80-1.32) | 0.853 | 0.956 | (0.60-1.53) | 0.854 | 0.951 | (0.63-1.44) | 0.819 |
|  | Eotaxin | 17 | 1.109 | (0.94-1.31) | 0.230 | 0.770 | (0.51-1.16) | 0.233 | 1.054 | (0.83-1.35) | 0.675 | 1.565 | (0.98-2.49) | 0.077 | 0.984 | (0.69-1.40) | 0.931 |
|  | GROa | 12 | 0.947 | (0.82-1.09) | 0.459 | 0.926 | (0.71-1.20) | 0.574 | 0.976 | (0.81-1.18) | 0.802 | 1.011 | (0.72-1.41) | 0.948 | 1.035 | (0.78-1.38) | 0.818 |
|  | IP10 | 12 | 1.011 | (0.87-1.18) | 0.890 | 1.067 | (0.75-1.52) | 0.725 | 0.971 | (0.79-1.19) | 0.780 | 0.836 | (0.59-1.19) | 0.340 | 0.844 | (0.59-1.20) | 0.367 |
|  | MCP1 | 16 | 0.924 | (0.76-1.12) | 0.417 | 1.095 | (0.65-1.85) | 0.741 | 0.901 | (0.70-1.16) | 0.414 | 0.859 | (0.56-1.32) | 0.497 | 0.869 | (0.59-1.28) | 0.491 |
|  | MCP3 | 6 | 1.035 | (0.90-1.19) | 0.626 | 0.835 | (0.60-1.17) | 0.352 | 1.031 | (0.87-1.21) | 0.718 | 0.925 | (0.69-1.24) | 0.623 | 0.939 | (0.71-1.25) | 0.684 |
|  | MIG | 11 | 1.074 | (0.89-1.29) | 0.452 | 0.931 | (0.65-1.34) | 0.708 | 1.011 | (0.81-1.26) | 0.918 | 0.980 | (0.68-1.41) | 0.915 | 1.003 | (0.70-1.43) | 0.987 |
|  | MIP1 | 4 | 0.892 | (0.75-1.06) | 0.203 | 0.960 | (0.54-1.70) | 0.901 | 0.898 | (0.73-1.10) | 0.300 | 0.902 | (0.68-1.20) | 0.534 | 0.906 | (0.69-1.19) | 0.523 |
|  | MIP1b | 22 | 1.151 | (0.98-1.35) | 0.090 | 1.168 | (0.73-1.87) | 0.526 | 1.065 | (0.85-1.33) | 0.579 | 1.081 | (0.72-1.62) | 0.708 | 1.037 | (0.69-1.56) | 0.864 |
|  | RANTES | 10 | 1.077 | (0.89-1.31) | 0.454 | 1.135 | (0.68-1.89) | 0.639 | 1.102 | (0.88-1.39) | 0.404 | 1.108 | (0.79-1.56) | 0.571 | 1.117 | (0.81-1.55) | 0.522 |
|  | SDF1a | 9 | 0.976 | (0.74-1.29) | 0.867 | 0.654 | (0.39-1.09) | 0.147 | 0.932 | (0.66-1.32) | 0.693 | 0.882 | (0.52-1.50) | 0.655 | 0.860 | (0.51-1.44) | 0.580 |
| **Growth factors** |  |  |  |  |  |  |  |  |  |  |  |  |  |  |  |  |  |
|  | bNGF | 4 | 1.006 | (0.81-1.24) | 0.957 | 0.483 | (0.18-1.31) | 0.389 | 0.998 | (0.77-1.30) | 0.989 | 0.931 | (0.68-1.28) | 0.704 | 0.978 | (0.71-1.34) | 0.904 |
|  | FGFBasic | 7 | 0.706 | (0.52-0.96) | **0.027** | 0.667 | (0.24-1.84) | 0.470 | 0.877 | (0.58-1.32) | 0.530 | 0.983 | (0.51-1.90) | 0.961 | 0.992 | (0.48-2.06) | 0.983 |
|  | GCSF | 9 | 0.907 | (0.63-1.30) | 0.597 | 1.186 | (0.64-2.21) | 0.609 | 0.999 | (0.69-1.46) | 0.997 | 0.912 | (0.46-1.80) | 0.797 | 0.981 | (0.57-1.68) | 0.946 |
|  | HGF | 9 | 0.904 | (0.67-1.23) | 0.517 | 1.121 | (0.56-2.26) | 0.759 | 1.026 | (0.71-1.49) | 0.890 | 1.071 | (0.61-1.90) | 0.819 | 1.186 | (0.74-1.90) | 0.501 |
|  | MCSF | 12 | 0.931 | (0.83-1.05) | 0.246 | 0.904 | (0.70-1.16) | 0.445 | 0.980 | (0.83-1.16) | 0.813 | 1.014 | (0.78-1.32) | 0.919 | 1.002 | (0.79-1.27) | 0.990 |
|  | PDGFbbc | 14 | 0.945 | (0.75-1.20) | 0.642 | 1.246 | (0.71-2.18) | 0.458 | 0.858 | (0.63-1.17) | 0.330 | 0.690 | (0.40-1.18) | 0.198 | 0.690 | (0.39-1.22) | 0.226 |
|  | SCF | 10 | 0.838 | (0.61-1.16) | 0.283 | 1.058 | (0.53-2.13) | 0.879 | 0.959 | (0.66-1.39) | 0.823 | 1.132 | (0.57-2.26) | 0.734 | 1.118 | (0.56-2.25) | 0.762 |
|  | SCGFbc | 21 | 1.061 | (0.95-1.18) | 0.289 | 1.094 | (0.87-1.37) | 0.449 | 1.068 | (0.92-1.24) | 0.401 | 1.072 | (0.84-1.37) | 0.590 | 1.097 | (0.87-1.38) | 0.446 |
|  | VEGFc | 18 | 0.941 | (0.81-1.10) | 0.438 | 0.803 | (0.57-1.13) | 0.221 | 0.976 | (0.78-1.22) | 0.831 | 1.075 | (0.75-1.54) | 0.700 | 1.037 | (0.74-1.46) | 0.836 |
| **Interleukins** |  |  |  |  |  |  |  |  |  |  |  |  |  |  |  |  |  |
|  | IL-10 | 15 | 0.930 | (0.75-1.16) | 0.514 | 0.525 | (0.27-1.01) | 0.075 | 0.817 | (0.63-1.06) | 0.123 | 1.038 | (0.61-1.77) | 0.892 | 0.840 | (0.61-1.16) | 0.311 |
|  | IL-12p70 | 15 | 1.019 | (0.84-1.24) | 0.848 | 1.483 | (0.73-3.02) | 0.299 | 1.176 | (0.89-1.55) | 0.250 | 1.363 | (0.82-2.26) | 0.249 | 1.329 | (0.85-2.08) | 0.234 |
|  | IL-13 | 14 | 0.949 | (0.83-1.08) | 0.434 | 0.733 | (0.57-0.94) | 0.028 | 0.911 | (0.76-1.10) | 0.331 | 0.891 | (0.66-1.20) | 0.464 | 0.906 | (0.68-1.21) | 0.510 |
|  | IL-16 | 10 | 1.021 | (0.92-1.13) | 0.682 | 0.979 | (0.83-1.16) | 0.812 | 1.032 | (0.92-1.16) | 0.593 | 0.952 | (0.75-1.21) | 0.696 | 1.070 | (0.94-1.22) | 0.352 |
|  | IL-17 | 8 | 0.817 | (0.65-1.03) | 0.087 | 0.820 | (0.53-1.26) | 0.402 | 0.831 | (0.61-1.13) | 0.243 | 0.752 | (0.48-1.17) | 0.247 | 0.847 | (0.58-1.24) | 0.421 |
|  | IL-18 | 13 | 0.936 | (0.76-1.15) | 0.524 | 1.081 | (0.73-1.61) | 0.708 | 0.871 | (0.71-1.07) | 0.196 | 0.864 | (0.63-1.19) | 0.387 | 0.856 | (0.61-1.20) | 0.382 |
|  | IL1b | 3 | 0.945 | (0.67-1.32) | 0.743 | 0.895 | (0.45-1.76) | 0.802 | 0.978 | (0.67-1.43) | 0.908 | 0.978 | (0.63-1.53) | 0.930 | 0.978 | (0.63-1.53) | 0.930 |
|  | IL1-ra | 10 | 1.103 | (0.91-1.33) | 0.308 | 1.512 | (0.91-2.51) | 0.149 | 1.008 | (0.80-1.28) | 0.950 | 0.991 | (0.66-1.49) | 0.968 | 0.991 | (0.68-1.45) | 0.966 |
|  | IL-2 | 7 | 1.179 | (1.01-1.38) | **0.041** | 1.108 | (0.67-1.83) | 0.705 | 1.190 | (1.01-1.40) | 0.036 | 0.963 | (0.65-1.42) | 0.855 | 1.017 | (0.76-1.36) | 0.915 |
|  | IL2-ra | 9 | 1.094 | (0.89-1.34) | 0.385 | 1.299 | (0.89-1.89) | 0.215 | 1.073 | (0.86-1.34) | 0.528 | 1.259 | (0.89-1.78) | 0.227 | 1.095 | (0.82-1.46) | 0.551 |
|  | IL-4 | 14 | 0.885 | (0.71-1.11) | 0.289 | 0.811 | (0.50-1.32) | 0.418 | 0.944 | (0.70-1.28) | 0.709 | 1.133 | (0.61-2.10) | 0.698 | 1.158 | (0.67-1.99) | 0.604 |
|  | IL-5 | 8 | 1.031 | (0.91-1.17) | 0.643 | 1.054 | (0.79-1.41) | 0.735 | 1.054 | (0.89-1.24) | 0.536 | 1.104 | (0.84-1.44) | 0.492 | 1.102 | (0.84-1.44) | 0.502 |
|  | IL-6 | 11 | 0.769 | (0.57-1.03) | 0.083 | 0.551 | (0.28-1.08) | 0.115 | 0.803 | (0.57-1.14) | 0.219 | 0.809 | (0.45-1.46) | 0.497 | 0.826 | (0.46-1.49) | 0.542 |
|  | IL-7 | 14 | 0.958 | (0.88-1.05) | 0.332 | 0.981 | (0.81-1.18) | 0.847 | 0.995 | (0.89-1.12) | 0.932 | 1.002 | (0.82-1.22) | 0.984 | 1.002 | (0.86-1.16) | 0.979 |
|  | IL-8 | 8 | 1.002 | (0.91-1.11) | 0.965 | 0.943 | (0.81-1.10) | 0.474 | 1.007 | (0.88-1.15) | 0.913 | 0.950 | (0.79-1.14) | 0.603 | 0.995 | (0.86-1.15) | 0.951 |
|  | IL-9 | 6 | 1.043 | (0.82-1.32) | 0.730 | 0.991 | (0.56-1.77) | 0.977 | 1.054 | (0.79-1.41) | 0.723 | 1.056 | (0.70-1.60) | 0.808 | 1.047 | (0.70-1.58) | 0.836 |
| **Others** |  |  |  |  |  |  |  |  |  |  |  |  |  |  |  |  |  |
|  | IFNg | 12 | 1.016 | (0.81-1.27) | 0.889 | 1.064 | (0.66-1.71) | 0.802 | 0.980 | (0.74-1.31) | 0.892 | 1.006 | (0.65-1.55) | 0.979 | 1.001 | (0.65-1.55) | 0.995 |
|  | MIF | 10 | 1.231 | (1.04-1.46) | **0.018** | 1.231 | (0.88-1.72) | 0.261 | 1.118 | (0.89-1.41) | 0.343 | 1.094 | (0.79-1.52) | 0.601 | 1.075 | (0.78-1.49) | 0.674 |
|  | TNFa | 4 | 1.034 | (0.74-1.44) | 0.844 | 0.744 | (0.49-1.12) | 0.293 | 0.900 | (0.65-1.24) | 0.522 | 0.868 | (0.57-1.32) | 0.557 | 0.866 | (0.58-1.30) | 0.536 |
|  | TNFb | 5 | 1.007 | (0.87-1.16) | 0.921 | 1.046 | (0.78-1.40) | 0.783 | 1.049 | (0.88-1.26) | 0.605 | 1.092 | (0.85-1.41) | 0.537 | 1.081 | (0.85-1.37) | 0.551 |
|  | TRAIL | 16 | 0.943 | (0.82-1.08) | 0.391 | 0.914 | (0.75-1.11) | 0.382 | 0.868 | (0.72-1.05) | 0.142 | 0.857 | (0.63-1.17) | 0.343 | 0.869 | (0.72-1.06) | 0.179 |

**Table S2.** Heterogeneity and horizontal pleiotropy tests of forty-one inflammatory cytokines on Parkinson’s disease.

| **Exposures** | **Q_1_ pval** | **Q_2_ pval** | **intercept** | **intercept pval** | **Recommended Method** |
| --- | --- | --- | --- | --- | --- |
| bNGF | 0.801 | 0.636 | 0.028 | 0.788 | IVW |
| CTACK | 0.070 | 0.030 | 0.070 | 0.152 | IVW |
| Eotaxin | 0.659 | 0.462 | 0.049 | 0.077 | IVW |
| FGFBasic | 0.258 | 0.364 | 0.0075 | 0.912 | IVW |
| GCSF | 0.027 | 0.035 | -0.042 | 0.337 | MR-Egger |
| GROa | 0.615 | 0.529 | 0.007 | 0.843 | IVW |
| HGF | 0.205 | 0.174 | -0.030 | 0.522 | IVW |
| IFNg | 0.358 | 0.284 | -0.007 | 0.829 | IVW |
| IL-10 | 0.067 | 0.154 | 0.066 | 0.094 | IVW |
| IL-12p70 | 0.470 | 0.481 | -0.040 | 0.303 | IVW |
| IL-13 | 0.477 | 0.883 | 0.065 | 0.030 | IVW |
| IL-16 | 0.109 | 0.090 | 0.015 | 0.538 | IVW |
| IL-17 | 0.967 | 0.932 | -7.20E-04 | 0.984 | IVW |
| IL-18 | 0.020 | 0.020 | -0.030 | 0.424 | MR-Egger |
| IL1b | 0.891 | 0.657 | 0.011 | 0.885 | IVW |
| IL1ra | 0.295 | 0.356 | -0.050 | 0.229 | IVW |
| IL-2 | 0.117 | 0.070 | -0.003 | 0.947 | IVW |
| IL-2ra | 0.096 | 0.114 | -0.037 | 0.324 | IVW |
| IL-4 | 0.312 | 0.256 | 0.012 | 0.697 | IVW |
| IL-5 | 0.663 | 0.765 | -0.005 | 0.874 | IVW |
| IL-6 | 0.144 | 0.125 | 0.040 | 0.307 | IVW |
| IL-7 | 0.821 | 0.763 | -0.006 | 0.780 | IVW |
| IL-8 | 0.817 | 0.857 | 0.018 | 0.341 | IVW |
| IL-9 | 0.930 | 0.860 | 0.013 | 0.859 | IVW |
| IP10 | 0.813 | 0.752 | -0.010 | 0.744 | IVW |
| MCP1 | 0.953 | 0.946 | -0.023 | 0.509 | IVW |
| MCP3 | 0.263 | 0.350 | 0.060 | 0.247 | IVW |
| MCSF | 0.561 | 0.477 | 0.010 | 0.791 | IVW |
| MIG | 0.172 | 0.170 | 0.036 | 0.389 | IVW |
| MIF | 0.789 | 0.704 | -1.01E-05 | 0.987 | IVW |
| MIP1a | 0.578 | 0.386 | -0.012 | 0.816 | IVW |
| MIP1b | 0.301 | 0.250 | -0.002 | 0.949 | IVW |
| PDGFbb | 0.222 | 0.236 | -0.034 | 0.309 | IVW |
| RANTES | 0.218 | 0.158 | -0.011 | 0.830 | IVW |
| SCF | 0.095 | 0.084 | -0.034 | 0.479 | IVW |
| SCGFb | 0.691 | 0.636 | -0.007 | 0.767 | IVW |
| SDF1a | 0.291 | 0.482 | 0.054 | 0.120 | IVW |
| TNFa | 0.188 | 0.724 | 0.096 | 0.179 | IVW |
| TNFb | 0.536 | 0.384 | -0.012 | 0.791 | IVW |
| TRAIL | 0.551 | 0.489 | 0.007 | 0.672 | IVW |
| VEGF | 0.747 | 0.760 | 0.026 | 0.317 | IVW |

Abbreviations: pval, p-value; Q, Cochran Q statistics; SNPs, single nucleotide polymorphisms; IVW, the inverse variance weighted method; Q_1_ pval: p value of Q test from IVW method; Q_2_ pval: p value of Q test from MR-Egger method

**Table S3.** SNPs information of forty-one inflammation cytokines with Parkinson’s disease.

| **Inflammatory cytokines** | | | | | | | **Parkinson’s disease** | |
| --- | --- | --- | --- | --- | --- | --- | --- | --- |
| **SNP** | **effect allele** | **other allele** | **Beta** | **se** | **pval** | **F** | **se** | **pval** |
| **bNGF** |  |  |  |  |  |  |  |  |
| rs28637706 | T | G | -0.155 | 0.026 | 2.72E-09 | 35.43 | 0.034 | 0.671 |
| rs4767014 | T | C | -0.121 | 0.026 | 4.52E-06 | 21.03 | 0.033 | 0.776 |
| rs71641308 | T | C | 0.197 | 0.043 | 4.42E-06 | 21.05 | 0.053 | 0.357 |
| rs73472576 | T | C | -0.115 | 0.025 | 4.81E-06 | 20.83 | 0.032 | 0.356 |
| **CTACK** |  |  |  |  |  |  |  |  |
| rs10854859 | A | G | -0.150 | 0.029 | 3.05E-07 | 26.12 | 0.038 | 0.428 |
| rs116303454 | A | G | 0.375 | 0.081 | 3.58E-06 | 21.47 | 0.099 | 0.291 |
| rs116871507 | A | T | -0.209 | 0.045 | 3.16E-06 | 21.67 | 0.062 | 0.024 |
| rs116943377 | A | G | 0.288 | 0.061 | 2.50E-06 | 22.17 | 0.082 | 0.094 |
| rs117932939 | T | C | 0.197 | 0.042 | 3.09E-06 | 21.76 | 0.057 | 0.946 |
| rs118084576 | A | G | 0.568 | 0.123 | 3.66E-06 | 21.41 | 0.179 | 0.082 |
| rs184329319 | T | G | -0.307 | 0.065 | 2.17E-06 | 22.42 | 0.087 | 0.571 |
| rs55764737 | T | C | 0.542 | 0.097 | 2.01E-08 | 31.44 | 0.132 | 0.266 |
| rs57338032 | A | G | 0.144 | 0.032 | 4.83E-06 | 20.84 | 0.042 | 0.020 |
| rs57789542 | T | C | -0.769 | 0.166 | 3.58E-06 | 21.46 | 0.196 | 0.994 |
| rs60247384 | T | C | 0.113 | 0.025 | 4.30E-06 | 21.19 | 0.033 | 0.044 |
| rs76395525 | A | G | 0.519 | 0.108 | 1.55E-06 | 23.06 | 0.156 | 0.716 |
| **Eotaxin** |  |  |  |  |  |  |  |  |
| rs11087905 | A | C | 0.095 | 0.033 | 4.07E-07 | 25.74 | 0.019 | 0.121 |
| rs112347425 | T | C | 0.160 | 0.055 | 7.77E-09 | 33.39 | 0.028 | 0.097 |
| rs11920996 | T | C | 0.298 | 0.074 | 2.92E-15 | 62.42 | 0.038 | 0.711 |
| rs1677588 | T | G | 0.118 | 0.050 | 2.22E-06 | 22.31 | 0.025 | 0.434 |
| rs2024050 | A | G | 0.164 | 0.061 | 5.47E-08 | 29.48 | 0.030 | 0.740 |
| rs2027855 | T | C | 0.074 | 0.032 | 4.27E-06 | 21.03 | 0.016 | 0.294 |
| rs2040143 | A | G | -0.086 | 0.035 | 1.33E-06 | 23.23 | 0.018 | 0.450 |
| rs2229593 | T | C | 0.365 | 0.080 | 2.84E-19 | 80.67 | 0.041 | 0.740 |
| rs2249581 | T | C | -0.090 | 0.036 | 5.91E-07 | 24.94 | 0.018 | 0.231 |
| rs5754733 | A | C | -0.105 | 0.042 | 8.20E-07 | 24.29 | 0.021 | 0.831 |
| rs57723662 | C | G | -0.098 | 0.042 | 3.88E-06 | 21.25 | 0.021 | 0.111 |
| rs60075014 | T | C | -0.169 | 0.064 | 2.08E-06 | 22.48 | 0.036 | 0.310 |
| rs7231030 | A | C | 0.090 | 0.038 | 2.71E-06 | 21.89 | 0.019 | 0.534 |
| rs73072941 | A | T | -0.128 | 0.049 | 1.11E-06 | 23.72 | 0.026 | 0.931 |
| rs745331 | A | G | -0.082 | 0.034 | 3.04E-06 | 21.75 | 0.018 | 0.216 |
| rs75426604 | A | C | -0.137 | 0.055 | 2.40E-06 | 22.19 | 0.029 | 0.131 |
| rs9317045 | A | C | 0.117 | 0.045 | 6.95E-07 | 24.66 | 0.024 | 0.615 |
| **GROa** |  |  |  |  |  |  |  |  |
| rs114991247 | T | C | -0.220 | 0.046 | 1.97E-06 | 22.61 | 0.053 | 0.910 |
| rs115214168 | T | C | 0.453 | 0.083 | 4.48E-08 | 29.89 | 0.108 | 0.778 |
| rs1361829 | A | G | -0.111 | 0.024 | 4.58E-06 | 21.05 | 0.032 | 0.935 |
| rs140734053 | A | G | 0.733 | 0.155 | 2.07E-06 | 22.51 | 0.169 | 0.441 |
| rs150194856 | T | C | -0.422 | 0.091 | 3.86E-06 | 21.34 | 0.121 | 0.616 |
| rs17171245 | T | G | 0.245 | 0.053 | 3.93E-06 | 21.29 | 0.074 | 0.657 |
| rs185768063 | A | G | 0.404 | 0.076 | 1.06E-07 | 28.21 | 0.096 | 0.703 |
| rs3026943 | A | C | -0.125 | 0.026 | 1.08E-06 | 23.68 | 0.033 | 0.821 |
| rs62024303 | A | G | -0.301 | 0.066 | 4.91E-06 | 20.83 | 0.080 | 0.259 |
| rs76215157 | C | G | -0.740 | 0.156 | 2.23E-06 | 22.36 | 0.164 | 0.011 |
| rs76390238 | C | G | 0.622 | 0.135 | 4.14E-06 | 21.17 | 0.170 | 0.479 |
| rs79454658 | T | C | 0.278 | 0.060 | 3.02E-06 | 21.81 | 0.076 | 0.758 |
| **GCSF** |  |  |  |  |  |  |  |  |
| rs10939033 | A | G | -0.078 | 0.016 | 2.07E-06 | 22.60 | 0.032 | 0.185 |
| rs117261691 | T | C | 0.132 | 0.029 | 4.67E-06 | 20.94 | 0.057 | 0.006 |
| rs183023730 | T | G | 0.790 | 0.168 | 2.47E-06 | 22.17 | 0.249 | 0.058 |
| rs586802 | A | G | 0.088 | 0.019 | 2.40E-06 | 22.24 | 0.037 | 0.726 |
| rs6740648 | T | C | 0.082 | 0.017 | 1.90E-06 | 22.61 | 0.034 | 0.679 |
| rs74148555 | T | C | -0.377 | 0.075 | 5.59E-07 | 25.07 | 0.111 | 0.876 |
| rs76287671 | T | C | 0.089 | 0.019 | 2.19E-06 | 22.37 | 0.037 | 0.985 |
| rs77318030 | T | C | -0.203 | 0.043 | 2.02E-06 | 22.62 | 0.078 | 0.208 |
| rs78523761 | A | G | 0.537 | 0.114 | 2.39E-06 | 22.25 | 0.206 | 0.075 |
| **FGFBasic** |  |  |  |  |  |  |  |  |
| rs13412535 | A | G | -0.113 | 0.022 | 4.76E-07 | 25.40 | 0.040 | 0.16 |
| rs147409637 | T | C | 0.201 | 0.043 | 3.08E-06 | 21.74 | 0.084 | 0.79 |
| rs17094040 | T | C | 0.105 | 0.023 | 4.31E-06 | 21.06 | 0.045 | 0.46 |
| rs2849358 | A | G | 0.091 | 0.019 | 2.32E-06 | 22.27 | 0.036 | 0.84 |
| rs4795091 | A | G | 0.124 | 0.027 | 3.06E-06 | 21.69 | 0.051 | 0.77 |
| rs76253061 | T | C | -0.481 | 0.104 | 3.81E-06 | 21.35 | 0.204 | 0.57 |
| rs78873483 | A | G | 0.129 | 0.028 | 4.98E-06 | 20.79 | 0.050 | 0.42 |
| **HGF** |  |  |  |  |  |  |  |  |
| rs11060254 | A | G | -0.077 | 0.017 | 3.97E-06 | 21.23 | 0.034 | 0.883 |
| rs11129909 | T | C | -0.074 | 0.016 | 4.46E-06 | 21.01 | 0.033 | 0.822 |
| rs2003620 | T | C | 0.228 | 0.049 | 2.98E-06 | 21.86 | 0.096 | 0.602 |
| rs362307 | T | C | 0.151 | 0.033 | 4.21E-06 | 21.22 | 0.063 | 0.698 |
| rs4245058 | T | C | -0.155 | 0.033 | 2.68E-06 | 21.98 | 0.065 | 0.940 |
| rs57146176 | A | G | -0.099 | 0.021 | 2.18E-06 | 22.51 | 0.063 | 0.707 |
| rs5745687 | T | C | -0.301 | 0.040 | 9.92E-14 | 55.42 | 0.079 | 0.193 |
| rs80051150 | T | C | 0.198 | 0.041 | 1.68E-06 | 22.98 | 0.086 | 0.067 |
| **IFNg** |  |  |  |  |  |  |  |  |
| rs113399544 | A | G | -0.085 | 0.018 | 3.32E-06 | 21.52 | 0.035 | 0.726 |
| rs113600793 | A | C | 0.187 | 0.037 | 4.43E-07 | 25.43 | 0.068 | 0.907 |
| rs115729819 | A | G | 0.251 | 0.051 | 1.05E-06 | 23.86 | 0.097 | 0.369 |
| rs117046255 | T | C | -0.097 | 0.021 | 2.79E-06 | 21.86 | 0.040 | 0.139 |
| rs11843756 | T | G | 0.181 | 0.039 | 3.62E-06 | 21.47 | 0.077 | 0.921 |
| rs12420286 | T | C | 0.236 | 0.050 | 2.45E-06 | 22.22 | 0.097 | 0.737 |
| rs147378920 | A | G | -0.384 | 0.075 | 3.20E-07 | 26.14 | 0.109 | 0.875 |
| rs1867282 | T | C | 0.078 | 0.017 | 2.48E-06 | 22.13 | 0.032 | 0.573 |
| rs2073438 | A | G | 0.092 | 0.019 | 9.55E-07 | 23.94 | 0.036 | 0.039 |
| rs7088799 | T | G | -0.081 | 0.017 | 1.27E-06 | 23.51 | 0.032 | 0.105 |
| rs73479333 | C | G | -0.112 | 0.024 | 2.82E-06 | 21.89 | 0.047 | 0.207 |
| rs74148555 | T | C | -0.377 | 0.077 | 9.86E-07 | 23.98 | 0.111 | 0.876 |
| **IL10** |  |  |  |  |  |  |  |  |
| rs10457128 | A | G | -0.085 | 0.017 | 6.96E-07 | 24.65 | 0.033 | 0.059 |
| rs10493718 | A | C | -0.108 | 0.022 | 1.07E-06 | 23.70 | 0.043 | 0.393 |
| rs13412535 | A | G | -0.135 | 0.022 | 1.80E-09 | 36.15 | 0.040 | 0.016 |
| rs1530455 | T | C | 0.082 | 0.017 | 2.53E-06 | 22.20 | 0.033 | 0.121 |
| rs2086656 | T | C | -0.080 | 0.017 | 2.59E-06 | 22.14 | 0.033 | 0.752 |
| rs3002131 | C | G | 0.119 | 0.026 | 4.59E-06 | 20.98 | 0.046 | 0.842 |
| rs3025021 | T | C | 0.091 | 0.019 | 2.61E-06 | 22.14 | 0.034 | 0.230 |
| rs383684 | A | G | 0.092 | 0.020 | 3.17E-06 | 21.80 | 0.055 | 0.482 |
| rs4741748 | A | G | -0.079 | 0.017 | 3.20E-06 | 21.74 | 0.032 | 0.360 |
| rs6054847 | T | C | 0.097 | 0.021 | 2.75E-06 | 22.00 | 0.040 | 0.627 |
| rs6680918 | T | C | -0.120 | 0.025 | 1.59E-06 | 23.11 | 0.048 | 0.912 |
| rs7088799 | T | G | -0.082 | 0.017 | 9.35E-07 | 24.10 | 0.032 | 0.105 |
| rs73192842 | A | G | 0.095 | 0.021 | 4.03E-06 | 21.22 | 0.040 | 0.900 |
| rs7747448 | A | G | -0.106 | 0.019 | 2.00E-08 | 31.51 | 0.036 | 0.078 |
| rs9472173 | T | C | -0.200 | 0.017 | 1.26E-30 | 132.61 | 0.032 | 0.168 |
| **IL12p70** |  |  |  |  |  |  |  |  |
| rs113600793 | A | C | 0.183 | 0.036 | 3.35E-07 | 26.03 | 0.068 | 0.907 |
| rs12969892 | T | C | 0.123 | 0.027 | 4.19E-06 | 21.11 | 0.052 | 0.264 |
| rs2123852 | T | C | 0.094 | 0.020 | 3.73E-06 | 21.32 | 0.039 | 0.579 |
| rs273702 | A | G | -0.127 | 0.027 | 2.52E-06 | 22.12 | 0.052 | 0.393 |
| rs282258 | T | C | 0.073 | 0.016 | 3.28E-06 | 21.65 | 0.032 | 0.534 |
| rs34322762 | T | C | 0.095 | 0.020 | 1.71E-06 | 22.93 | 0.033 | 0.515 |
| rs34826779 | T | G | -0.088 | 0.019 | 3.33E-06 | 21.64 | 0.038 | 0.164 |
| rs41282644 | A | G | 0.140 | 0.030 | 3.74E-06 | 21.37 | 0.057 | 0.267 |
| rs4530855 | T | G | 0.086 | 0.018 | 2.87E-06 | 21.89 | 0.035 | 0.380 |
| rs4741748 | A | G | -0.080 | 0.016 | 9.16E-07 | 24.02 | 0.032 | 0.360 |
| rs6532374 | T | C | -0.103 | 0.023 | 4.61E-06 | 20.89 | 0.045 | 0.063 |
| rs7754905 | A | G | -0.101 | 0.019 | 1.14E-07 | 27.97 | 0.038 | 0.438 |
| rs782111 | A | C | -0.077 | 0.016 | 9.25E-07 | 24.04 | 0.032 | 0.321 |
| rs865585 | A | C | -0.165 | 0.024 | 2.73E-12 | 48.69 | 0.045 | 0.396 |
| rs9381249 | T | C | -0.179 | 0.037 | 1.13E-06 | 23.73 | 0.079 | 0.748 |
| **IL13** |  |  |  |  |  |  |  |  |
| rs10995604 | A | G | -0.157 | 0.034 | 4.48E-06 | 20.97 | 0.044 | 0.685 |
| rs117795020 | A | G | -0.358 | 0.072 | 5.48E-07 | 25.04 | 0.093 | 0.188 |
| rs12623722 | A | G | -0.119 | 0.026 | 3.61E-06 | 21.39 | 0.034 | 0.125 |
| rs138854806 | A | G | -0.420 | 0.084 | 5.45E-07 | 25.09 | 0.093 | 0.553 |
| rs139083458 | T | C | 1.000 | 0.211 | 2.17E-06 | 22.43 | 0.279 | 0.494 |
| rs147747784 | C | G | 0.369 | 0.077 | 1.44E-06 | 23.25 | 0.074 | 0.861 |
| rs150836197 | T | C | 0.328 | 0.071 | 4.14E-06 | 21.19 | 0.080 | 0.696 |
| rs27949 | T | C | -0.114 | 0.025 | 4.83E-06 | 20.93 | 0.034 | 0.219 |
| rs28442067 | A | G | -0.138 | 0.029 | 1.41E-06 | 23.24 | 0.038 | 0.311 |
| rs7073807 | T | C | 0.162 | 0.035 | 4.77E-06 | 20.88 | 0.046 | 0.603 |
| rs75383097 | C | G | -0.537 | 0.116 | 3.70E-06 | 21.41 | 0.127 | 0.067 |
| rs76339001 | A | T | -0.438 | 0.089 | 7.92E-07 | 24.37 | 0.094 | 0.921 |
| rs76975337 | T | C | -0.121 | 0.027 | 4.92E-06 | 20.87 | 0.036 | 0.682 |
| rs77955971 | A | C | 0.441 | 0.087 | 3.76E-07 | 25.78 | 0.087 | 0.201 |
| **IL16** |  |  |  |  |  |  |  |  |
| rs117217798 | T | C | -0.206 | 0.044 | 2.77E-06 | 21.99 | 0.048 | 0.999 |
| rs12577604 | T | C | 0.434 | 0.094 | 4.08E-06 | 21.21 | 0.097 | 0.117 |
| rs142034902 | A | G | -0.437 | 0.093 | 2.33E-06 | 22.28 | 0.063 | 0.253 |
| rs142332135 | A | G | -0.765 | 0.108 | 1.58E-12 | 49.91 | 0.110 | 0.781 |
| rs144691581 | A | G | 0.493 | 0.096 | 2.67E-07 | 26.46 | 0.109 | 0.293 |
| rs35834666 | T | C | -0.173 | 0.035 | 6.57E-07 | 24.67 | 0.026 | 0.050 |
| rs4778640 | A | G | 0.719 | 0.098 | 2.55E-13 | 53.45 | 0.047 | 0.138 |
| rs4976691 | C | G | 0.125 | 0.026 | 1.47E-06 | 23.25 | 0.022 | 0.275 |
| rs7097884 | T | C | -0.119 | 0.024 | 8.81E-07 | 24.09 | 0.020 | 0.688 |
| rs78042619 | A | G | 0.550 | 0.116 | 2.02E-06 | 22.55 | 0.057 | 0.127 |
| **IL17** |  |  |  |  |  |  |  |  |
| rs11985957 | A | G | 0.151 | 0.033 | 4.36E-06 | 21.09 | 0.068 | 0.241 |
| rs12735700 | T | G | -0.094 | 0.021 | 4.50E-06 | 20.95 | 0.039 | 0.767 |
| rs145006174 | C | G | -0.227 | 0.047 | 1.65E-06 | 22.94 | 0.091 | 0.580 |
| rs17282552 | T | C | -0.203 | 0.040 | 4.88E-07 | 25.27 | 0.070 | 0.416 |
| rs3792369 | A | G | 0.094 | 0.017 | 1.46E-08 | 32.13 | 0.032 | 0.287 |
| rs61990749 | C | G | 0.112 | 0.023 | 6.57E-07 | 24.73 | 0.044 | 0.852 |
| rs78296352 | T | G | 0.295 | 0.065 | 4.81E-06 | 20.90 | 0.134 | 0.383 |
| rs9519328 | A | G | 0.526 | 0.110 | 1.79E-06 | 22.78 | 0.104 | 0.518 |
| **IL18** |  |  |  |  |  |  |  |  |
| rs10409850 | A | G | 0.179 | 0.035 | 2.44E-07 | 26.63 | 0.047 | 0.457 |
| rs11214093 | T | C | 0.114 | 0.024 | 1.54E-06 | 23.05 | 0.032 | 0.807 |
| rs117266781 | T | C | 0.705 | 0.144 | 9.18E-07 | 24.10 | 0.170 | 0.019 |
| rs117371668 | T | G | 0.371 | 0.080 | 3.36E-06 | 21.57 | 0.110 | 0.374 |
| rs139468359 | T | C | 0.510 | 0.109 | 2.74E-06 | 21.97 | 0.143 | 0.962 |
| rs1979967 | T | C | 0.140 | 0.029 | 8.72E-07 | 24.12 | 0.038 | 0.613 |
| rs4952239 | A | T | -0.116 | 0.024 | 1.81E-06 | 22.81 | 0.033 | 0.004 |
| rs58701153 | A | T | -0.127 | 0.024 | 1.81E-07 | 27.31 | 0.033 | 0.586 |
| rs62312914 | T | C | -0.127 | 0.025 | 4.22E-07 | 25.59 | 0.033 | 0.505 |
| rs764078 | A | T | 0.128 | 0.028 | 4.08E-06 | 21.29 | 0.037 | 0.962 |
| rs77187209 | T | C | -0.486 | 0.104 | 3.08E-06 | 21.77 | 0.132 | 0.274 |
| rs78623212 | T | C | 0.832 | 0.168 | 6.82E-07 | 24.64 | 0.181 | 0.089 |
| rs78716465 | A | G | 0.317 | 0.068 | 2.98E-06 | 21.83 | 0.085 | 0.029 |
| **IL1b** |  |  |  |  |  |  |  |  |
| rs143319329 | T | C | 0.436 | 0.093 | 2.84E-06 | 21.94 | 0.197 | 0.570 |
| rs4786740 | A | C | 0.126 | 0.027 | 1.82E-06 | 22.74 | 0.032 | 0.932 |
| rs61335305 | A | C | 0.433 | 0.093 | 3.02E-06 | 21.79 | 0.118 | 0.934 |
| **IL1ra** |  |  |  |  |  |  |  |  |
| rs1054402 | T | C | 0.133 | 0.027 | 8.20E-07 | 24.25 | 0.036 | 0.904 |
| rs117181659 | A | G | -0.220 | 0.048 | 3.92E-06 | 21.25 | 0.064 | 0.645 |
| rs11869294 | C | G | -0.229 | 0.047 | 1.13E-06 | 23.64 | 0.059 | 0.030 |
| rs13343438 | A | G | 0.277 | 0.061 | 4.97E-06 | 20.83 | 0.081 | 0.091 |
| rs35590641 | C | G | -0.117 | 0.025 | 3.04E-06 | 21.78 | 0.033 | 0.743 |
| rs3876037 | A | G | 0.123 | 0.027 | 4.73E-06 | 20.88 | 0.033 | 0.784 |
| rs56134659 | A | G | -0.111 | 0.024 | 2.56E-06 | 22.07 | 0.031 | 0.103 |
| rs61335305 | A | C | 0.432 | 0.090 | 1.81E-06 | 22.77 | 0.118 | 0.934 |
| rs6699436 | A | G | -0.186 | 0.040 | 4.37E-06 | 21.14 | 0.051 | 0.930 |
| rs9985296 | T | C | 0.105 | 0.023 | 4.95E-06 | 20.77 | 0.032 | 0.252 |
| **IL2** |  |  |  |  |  |  |  |  |
| rs13412535 | A | G | 0.174 | 0.033 | 1.45E-07 | 27.62 | 0.040 | 0.016 |
| rs16836080 | A | G | 0.116 | 0.025 | 4.84E-06 | 20.94 | 0.034 | 0.700 |
| rs4479767 | A | G | 0.182 | 0.039 | 3.44E-06 | 21.57 | 0.054 | 0.865 |
| rs4634519 | A | G | -0.125 | 0.027 | 3.18E-06 | 21.71 | 0.035 | 0.092 |
| rs61335305 | A | C | 0.444 | 0.091 | 1.16E-06 | 23.63 | 0.118 | 0.934 |
| rs62124990 | T | G | -0.701 | 0.149 | 2.50E-06 | 22.14 | 0.119 | 0.654 |
| rs7615304 | A | G | -0.114 | 0.024 | 2.16E-06 | 22.51 | 0.032 | 0.153 |
| **IL2ra** |  |  |  |  |  |  |  |  |
| rs11241559 | T | G | -0.124 | 0.026 | 2.75E-06 | 22.05 | 0.036 | 0.045 |
| rs117244812 | A | G | -0.719 | 0.149 | 1.47E-06 | 23.16 | 0.191 | 0.897 |
| rs12789243 | T | C | 0.126 | 0.028 | 4.61E-06 | 20.93 | 0.037 | 0.554 |
| rs17147986 | A | C | -0.298 | 0.034 | 1.07E-18 | 77.94 | 0.045 | 0.916 |
| rs17624670 | A | G | -0.125 | 0.027 | 4.64E-06 | 20.95 | 0.036 | 0.392 |
| rs34037190 | A | G | 0.478 | 0.094 | 3.13E-07 | 26.17 | 0.096 | 0.064 |
| rs56213152 | T | C | 0.127 | 0.027 | 2.94E-06 | 21.92 | 0.037 | 0.084 |
| rs759244 | A | T | -0.109 | 0.024 | 4.26E-06 | 21.12 | 0.032 | 0.159 |
| rs79100208 | C | G | 0.835 | 0.176 | 2.05E-06 | 22.52 | 0.209 | 0.263 |
| **IL4** |  |  |  |  |  |  |  |  |
| rs116705532 | T | G | -0.468 | 0.098 | 1.73E-06 | 22.84 | 0.194 | 0.270 |
| rs117146485 | T | C | -0.286 | 0.063 | 4.95E-06 | 20.88 | 0.118 | 0.721 |
| rs12238729 | T | C | 0.527 | 0.110 | 1.51E-06 | 23.12 | 0.262 | 0.995 |
| rs12640583 | T | G | -0.110 | 0.021 | 2.45E-07 | 26.61 | 0.042 | 0.110 |
| rs17713451 | A | G | 0.126 | 0.025 | 6.41E-07 | 24.80 | 0.050 | 0.225 |
| rs1867282 | T | C | 0.081 | 0.016 | 5.82E-07 | 24.87 | 0.032 | 0.573 |
| rs2073438 | A | G | 0.085 | 0.018 | 3.73E-06 | 21.42 | 0.036 | 0.039 |
| rs2346020 | A | G | 0.079 | 0.017 | 2.84E-06 | 21.85 | 0.034 | 0.389 |
| rs2708586 | T | C | -0.077 | 0.017 | 3.59E-06 | 21.34 | 0.033 | 0.508 |
| rs56408830 | A | G | -0.179 | 0.037 | 9.08E-07 | 24.15 | 0.075 | 0.107 |
| rs7613691 | A | G | 0.179 | 0.038 | 2.96E-06 | 21.88 | 0.073 | 0.162 |
| rs79597994 | T | C | -0.586 | 0.127 | 4.06E-06 | 21.22 | 0.191 | 0.491 |
| rs9506111 | A | G | -0.145 | 0.031 | 4.08E-06 | 21.20 | 0.062 | 0.913 |
| rs9941733 | A | G | 0.116 | 0.023 | 4.33E-07 | 25.48 | 0.042 | 0.822 |
| **IL5** |  |  |  |  |  |  |  |  |
| rs10178043 | T | G | 0.258 | 0.055 | 3.13E-06 | 21.74 | 0.044 | 0.424 |
| rs148634917 | A | G | -0.517 | 0.109 | 1.97E-06 | 22.61 | 0.073 | 0.375 |
| rs28793375 | T | C | 0.170 | 0.036 | 2.75E-06 | 21.96 | 0.032 | 0.766 |
| rs72831687 | A | G | -0.534 | 0.110 | 1.32E-06 | 23.36 | 0.135 | 0.347 |
| rs73040118 | T | C | 0.229 | 0.049 | 2.90E-06 | 21.90 | 0.042 | 0.477 |
| rs74811276 | A | G | 0.217 | 0.047 | 4.08E-06 | 21.21 | 0.045 | 0.811 |
| rs7739450 | A | G | -0.130 | 0.026 | 4.05E-07 | 25.57 | 0.026 | 0.629 |
| rs9309063 | T | G | -0.112 | 0.025 | 4.87E-06 | 20.85 | 0.023 | 0.286 |
| **IL6** |  |  |  |  |  |  |  |  |
| rs10910395 | A | T | -0.108 | 0.024 | 4.35E-06 | 21.12 | 0.046 | 0.714 |
| rs10982193 | A | G | -0.079 | 0.017 | 4.82E-06 | 20.77 | 0.035 | 0.597 |
| rs113098456 | A | G | -0.155 | 0.034 | 4.64E-06 | 20.98 | 0.061 | 0.002 |
| rs113600793 | A | C | 0.174 | 0.036 | 1.29E-06 | 23.38 | 0.068 | 0.907 |
| rs114373846 | T | C | 0.420 | 0.091 | 3.57E-06 | 21.49 | 0.188 | 0.320 |
| rs11732981 | A | C | 0.072 | 0.016 | 3.79E-06 | 21.42 | 0.031 | 0.584 |
| rs1333040 | T | C | 0.075 | 0.016 | 1.99E-06 | 22.63 | 0.031 | 0.216 |
| rs13412535 | A | G | -0.119 | 0.021 | 3.14E-08 | 30.71 | 0.040 | 0.016 |
| rs4684700 | T | C | -0.075 | 0.016 | 3.91E-06 | 21.26 | 0.032 | 0.480 |
| rs73273528 | T | C | 0.268 | 0.055 | 1.25E-06 | 23.48 | 0.105 | 0.786 |
| rs76856708 | T | C | 0.336 | 0.070 | 1.43E-06 | 23.23 | 0.145 | 0.463 |
| **IL7** |  |  |  |  |  |  |  |  |
| rs115215018 | T | C | 0.599 | 0.131 | 4.76E-06 | 20.92 | 0.095 | 0.356 |
| rs117509142 | T | C | -0.321 | 0.068 | 2.60E-06 | 22.05 | 0.072 | 0.892 |
| rs11757972 | T | C | 0.121 | 0.026 | 2.53E-06 | 22.15 | 0.023 | 0.422 |
| rs1374279 | A | T | 0.163 | 0.035 | 2.79E-06 | 21.92 | 0.034 | 0.330 |
| rs142397827 | A | C | 0.459 | 0.099 | 3.82E-06 | 21.33 | 0.075 | 0.985 |
| rs17091524 | T | C | 0.509 | 0.102 | 5.24E-07 | 25.15 | 0.073 | 0.532 |
| rs2006957 | T | C | 0.256 | 0.026 | 1.43E-22 | 95.19 | 0.024 | 0.890 |
| rs218238 | A | T | 0.132 | 0.028 | 3.28E-06 | 21.56 | 0.022 | 0.776 |
| rs28793375 | T | C | 0.164 | 0.036 | 4.87E-06 | 20.84 | 0.032 | 0.766 |
| rs62006410 | T | C | -0.149 | 0.030 | 7.59E-07 | 24.39 | 0.026 | 0.205 |
| rs7155170 | A | T | -0.124 | 0.027 | 4.79E-06 | 20.94 | 0.021 | 0.075 |
| rs77318030 | T | C | -0.297 | 0.063 | 2.64E-06 | 22.08 | 0.059 | 0.442 |
| rs77981494 | T | C | -0.520 | 0.106 | 8.23E-07 | 24.29 | 0.081 | 0.865 |
| **IL8** |  |  |  |  |  |  |  |  |
| rs113487695 | A | C | -0.613 | 0.129 | 2.09E-06 | 22.49 | 0.053 | 0.726 |
| rs116726256 | T | C | -0.225 | 0.049 | 4.26E-06 | 21.10 | 0.079 | 0.776 |
| rs12075 | A | G | 0.115 | 0.024 | 9.97E-07 | 23.85 | 0.017 | 0.963 |
| rs12912642 | A | G | 0.117 | 0.025 | 3.21E-06 | 21.64 | 0.024 | 0.405 |
| rs183628733 | T | C | 0.655 | 0.142 | 3.82E-06 | 21.34 | 0.072 | 0.796 |
| rs2673604 | A | C | -0.118 | 0.025 | 3.29E-06 | 21.57 | 0.024 | 0.229 |
| rs3786107 | A | G | 0.246 | 0.052 | 1.94E-06 | 22.68 | 0.054 | 0.711 |
| rs75840288 | A | C | 0.513 | 0.112 | 4.85E-06 | 20.89 | 0.063 | 0.292 |
| **IL9** |  |  |  |  |  |  |  |  |
| rs117807175 | C | G | -0.523 | 0.111 | 2.33E-06 | 22.31 | 0.143 | 0.967 |
| rs1259728 | A | G | -0.238 | 0.051 | 2.60E-06 | 22.04 | 0.068 | 0.530 |
| rs3736858 | C | G | -0.135 | 0.029 | 3.37E-06 | 21.54 | 0.039 | 0.849 |
| rs41294750 | T | C | 0.344 | 0.074 | 2.92E-06 | 21.86 | 0.095 | 0.849 |
| rs4880409 | T | C | -0.355 | 0.072 | 6.95E-07 | 24.60 | 0.153 | 0.844 |
| rs73443903 | A | C | 0.216 | 0.046 | 2.57E-06 | 22.08 | 0.062 | 0.328 |
| **IP10** |  |  |  |  |  |  |  |  |
| rs113183470 | A | T | -0.241 | 0.052 | 4.15E-06 | 21.21 | 0.067 | 0.478 |
| rs12714300 | A | T | -0.157 | 0.034 | 3.30E-06 | 21.65 | 0.045 | 0.501 |
| rs143799975 | A | G | -0.755 | 0.164 | 4.01E-06 | 21.24 | 0.194 | 0.543 |
| rs34383175 | T | C | -0.320 | 0.065 | 9.90E-07 | 23.94 | 0.088 | 0.295 |
| rs397816 | T | C | 0.121 | 0.025 | 1.03E-06 | 23.83 | 0.033 | 0.556 |
| rs4859940 | C | G | -0.120 | 0.026 | 3.23E-06 | 21.77 | 0.035 | 0.581 |
| rs4862110 | T | C | -0.145 | 0.032 | 4.91E-06 | 20.87 | 0.037 | 0.370 |
| rs75970138 | A | G | -0.485 | 0.104 | 2.99E-06 | 21.82 | 0.145 | 0.509 |
| rs7645625 | T | G | -0.112 | 0.024 | 2.19E-06 | 22.35 | 0.032 | 0.530 |
| rs78077394 | T | C | -0.349 | 0.070 | 6.47E-07 | 24.72 | 0.086 | 0.956 |
| rs79848609 | A | C | 0.251 | 0.054 | 2.64E-06 | 22.07 | 0.072 | 0.274 |
| rs8112618 | A | G | 0.139 | 0.030 | 3.05E-06 | 21.83 | 0.040 | 0.335 |
| **MCP1** |  |  |  |  |  |  |  |  |
| rs111995966 | T | G | 0.143 | 0.031 | 3.79E-06 | 21.35 | 0.059 | 0.610 |
| rs11920996 | T | C | 0.181 | 0.038 | 1.60E-06 | 23.04 | 0.074 | 0.711 |
| rs12062235 | T | G | 0.148 | 0.032 | 3.84E-06 | 21.30 | 0.065 | 0.823 |
| rs143815843 | A | G | -0.205 | 0.045 | 4.61E-06 | 21.01 | 0.093 | 0.644 |
| rs16837903 | A | G | -0.110 | 0.024 | 3.35E-06 | 21.51 | 0.046 | 0.149 |
| rs2201150 | T | C | 0.092 | 0.016 | 1.04E-08 | 32.77 | 0.032 | 0.751 |
| rs2229593 | T | C | 0.262 | 0.041 | 9.25E-11 | 41.97 | 0.080 | 0.740 |
| rs56212190 | T | C | 0.180 | 0.037 | 1.32E-06 | 23.38 | 0.073 | 0.673 |
| rs62245103 | T | G | 0.243 | 0.042 | 4.99E-09 | 34.20 | 0.077 | 0.812 |
| rs7197349 | A | G | 0.097 | 0.021 | 2.40E-06 | 22.21 | 0.039 | 0.281 |
| rs72705803 | A | G | -0.219 | 0.047 | 3.22E-06 | 21.67 | 0.098 | 0.500 |
| rs7978037 | A | T | 0.075 | 0.016 | 3.04E-06 | 21.73 | 0.032 | 0.881 |
| rs79939301 | A | G | 0.145 | 0.026 | 1.36E-08 | 32.28 | 0.050 | 0.541 |
| rs856100 | A | G | 0.090 | 0.019 | 2.55E-06 | 22.15 | 0.038 | 0.682 |
| rs862990 | T | C | -0.089 | 0.018 | 1.15E-06 | 23.65 | 0.036 | 0.141 |
| rs9317045 | A | C | 0.116 | 0.024 | 8.43E-07 | 24.23 | 0.045 | 0.615 |
| **MCP3** |  |  |  |  |  |  |  |  |
| rs10892381 | T | C | 0.243 | 0.047 | 2.69E-07 | 26.39 | 0.033 | 0.158 |
| rs117286643 | A | G | 0.693 | 0.147 | 2.54E-06 | 22.09 | 0.105 | 0.312 |
| rs28394764 | A | T | 0.597 | 0.128 | 3.19E-06 | 21.65 | 0.090 | 0.890 |
| rs3129806 | T | C | -0.198 | 0.043 | 4.98E-06 | 20.77 | 0.032 | 0.119 |
| rs6993671 | T | C | 0.204 | 0.044 | 4.06E-06 | 21.19 | 0.032 | 0.634 |
| rs7275485 | T | C | -0.222 | 0.048 | 3.99E-06 | 21.22 | 0.035 | 0.300 |
| **MCSF** |  |  |  |  |  |  |  |  |
| rs116274860 | T | G | 0.826 | 0.174 | 2.03E-06 | 22.55 | 0.141 | 0.666 |
| rs116887628 | A | G | -0.274 | 0.060 | 4.63E-06 | 20.99 | 0.062 | 0.081 |
| rs117867915 | T | C | 0.522 | 0.110 | 1.87E-06 | 22.70 | 0.112 | 0.989 |
| rs11963606 | C | G | -0.535 | 0.117 | 4.73E-06 | 20.92 | 0.126 | 0.244 |
| rs12962919 | T | C | 0.303 | 0.066 | 4.39E-06 | 21.05 | 0.063 | 0.960 |
| rs139457375 | A | C | -0.405 | 0.085 | 2.14E-06 | 22.44 | 0.090 | 0.257 |
| rs147378920 | A | G | -0.606 | 0.132 | 4.18E-06 | 21.15 | 0.109 | 0.875 |
| rs34089869 | T | C | 0.219 | 0.046 | 2.08E-06 | 22.53 | 0.050 | 0.748 |
| rs62294910 | A | G | 0.347 | 0.069 | 4.38E-07 | 25.52 | 0.065 | 0.834 |
| rs72723242 | T | G | -0.497 | 0.108 | 4.43E-06 | 21.03 | 0.124 | 0.108 |
| rs9387100 | T | C | -0.135 | 0.029 | 3.34E-06 | 21.65 | 0.032 | 0.558 |
| rs9626985 | T | C | 0.228 | 0.050 | 4.48E-06 | 21.06 | 0.054 | 0.154 |
| **MIF** |  |  |  |  |  |  |  |  |
| rs1007888 | T | C | -0.128 | 0.025 | 1.92E-07 | 27.07 | 0.032 | 0.283 |
| rs113218956 | A | G | -0.879 | 0.188 | 2.82E-06 | 21.94 | 0.242 | 0.305 |
| rs11551183 | C | G | 0.367 | 0.080 | 4.00E-06 | 21.25 | 0.107 | 0.758 |
| rs12594190 | A | G | 0.132 | 0.027 | 6.85E-07 | 24.65 | 0.034 | 0.979 |
| rs141009259 | T | C | -0.619 | 0.129 | 1.44E-06 | 23.22 | 0.148 | 0.202 |
| rs2294689 | C | G | -0.134 | 0.029 | 3.04E-06 | 21.72 | 0.131 | 0.895 |
| rs35792361 | A | G | -0.259 | 0.053 | 9.00E-07 | 24.07 | 0.067 | 0.787 |
| rs35890933 | T | G | 0.168 | 0.037 | 4.46E-06 | 21.07 | 0.045 | 0.009 |
| rs3814097 | A | G | -0.116 | 0.025 | 3.55E-06 | 21.46 | 0.032 | 0.685 |
| rs78098071 | T | C | -0.458 | 0.092 | 5.51E-07 | 25.07 | 0.122 | 0.945 |
| **MIG** |  |  |  |  |  |  |  |  |
| rs10266753 | T | C | -0.202 | 0.040 | 3.77E-07 | 25.77 | 0.056 | 0.088 |
| rs111607343 | A | G | -0.524 | 0.112 | 2.93E-06 | 21.87 | 0.138 | 0.044 |
| rs11177248 | A | G | 0.316 | 0.067 | 2.22E-06 | 22.39 | 0.082 | 0.394 |
| rs113302091 | T | C | 0.254 | 0.055 | 4.40E-06 | 21.04 | 0.075 | 0.489 |
| rs139010077 | T | C | 0.434 | 0.094 | 4.19E-06 | 21.14 | 0.119 | 0.953 |
| rs192433162 | A | G | -0.805 | 0.168 | 1.59E-06 | 23.03 | 0.210 | 0.943 |
| rs3733233 | T | C | 0.122 | 0.025 | 1.05E-06 | 23.92 | 0.033 | 0.031 |
| rs62562991 | A | G | 0.624 | 0.126 | 7.24E-07 | 24.54 | 0.134 | 0.612 |
| rs6679677 | A | C | 0.163 | 0.033 | 6.51E-07 | 24.77 | 0.044 | 0.701 |
| rs8127917 | T | G | 0.238 | 0.049 | 1.28E-06 | 23.43 | 0.066 | 0.879 |
| rs816960 | T | C | -0.118 | 0.024 | 1.15E-06 | 23.72 | 0.033 | 0.213 |
| **MIP1a** |  |  |  |  |  |  |  |  |
| rs117506943 | T | C | 0.313 | 0.068 | 4.48E-06 | 21.02 | 0.052 | 0.349 |
| rs12159394 | A | G | -0.171 | 0.037 | 3.11E-06 | 21.77 | 0.034 | 0.722 |
| rs57786342 | A | G | 0.139 | 0.028 | 8.91E-07 | 24.11 | 0.021 | 0.620 |
| rs6956239 | T | C | 0.119 | 0.026 | 4.58E-06 | 20.94 | 0.025 | 0.126 |
| **MIP1b** |  |  |  |  |  |  |  |  |
| rs111721971 | T | G | -0.227 | 0.047 | 1.39E-06 | 23.32 | 0.088 | 0.880 |
| rs116237296 | A | G | 0.528 | 0.112 | 2.15E-06 | 22.45 | 0.233 | 0.491 |
| rs11651720 | T | C | -0.119 | 0.022 | 5.69E-08 | 29.47 | 0.044 | 0.134 |
| rs11716293 | C | G | 0.099 | 0.019 | 1.69E-07 | 27.21 | 0.037 | 0.418 |
| rs117657747 | A | G | 0.209 | 0.045 | 4.01E-06 | 21.26 | 0.070 | 0.917 |
| rs145526037 | T | G | -0.186 | 0.041 | 4.47E-06 | 21.05 | 0.081 | 0.416 |
| rs17138331 | A | G | -0.143 | 0.030 | 1.13E-06 | 23.62 | 0.057 | 0.993 |
| rs17661219 | C | G | 0.087 | 0.017 | 3.89E-07 | 25.70 | 0.033 | 0.328 |
| rs2314809 | T | C | -0.074 | 0.016 | 2.90E-06 | 21.91 | 0.031 | 0.847 |
| rs2742396 | T | C | 0.107 | 0.017 | 1.83E-10 | 40.63 | 0.032 | 0.821 |
| rs281728 | A | C | -0.079 | 0.017 | 3.89E-06 | 21.34 | 0.034 | 0.252 |
| rs28393318 | A | G | -0.108 | 0.024 | 4.62E-06 | 20.96 | 0.048 | 0.883 |
| rs57893487 | C | G | -0.108 | 0.021 | 4.92E-07 | 25.23 | 0.039 | 0.002 |
| rs6802288 | A | G | -0.162 | 0.017 | 6.57E-21 | 87.99 | 0.035 | 0.524 |
| rs6806860 | A | C | -0.101 | 0.019 | 8.58E-08 | 28.74 | 0.038 | 0.085 |
| rs6908843 | A | G | 0.100 | 0.021 | 1.78E-06 | 22.75 | 0.042 | 0.182 |
| rs72791296 | T | C | 0.236 | 0.047 | 3.97E-07 | 25.73 | 0.088 | 0.976 |
| rs72799710 | T | C | -0.104 | 0.022 | 1.79E-06 | 22.83 | 0.043 | 0.942 |
| rs74979864 | A | T | -0.318 | 0.061 | 2.03E-07 | 26.97 | 0.121 | 0.919 |
| rs76582507 | A | G | 0.326 | 0.068 | 1.42E-06 | 23.24 | 0.174 | 0.701 |
| rs772112 | A | T | -0.142 | 0.022 | 5.01E-11 | 43.21 | 0.040 | 0.064 |
| rs9916627 | T | C | -0.096 | 0.020 | 1.24E-06 | 23.50 | 0.040 | 0.139 |
| **PDGFbb** |  |  |  |  |  |  |  |  |
| rs10512952 | T | C | -0.282 | 0.059 | 1.64E-06 | 23.01 | 0.118 | 0.399 |
| rs116154010 | T | C | 0.323 | 0.066 | 1.11E-06 | 23.73 | 0.134 | 0.043 |
| rs11766649 | A | G | 0.090 | 0.020 | 3.96E-06 | 21.17 | 0.039 | 0.800 |
| rs12289510 | A | G | -0.077 | 0.016 | 1.00E-06 | 23.87 | 0.032 | 0.144 |
| rs12615784 | T | C | -0.100 | 0.019 | 1.99E-07 | 27.00 | 0.039 | 0.566 |
| rs147862316 | T | C | 0.228 | 0.041 | 2.99E-08 | 30.74 | 0.084 | 0.181 |
| rs2643354 | A | G | 0.125 | 0.026 | 1.63E-06 | 22.97 | 0.053 | 0.252 |
| rs35859699 | A | G | -0.385 | 0.084 | 4.22E-06 | 21.15 | 0.155 | 0.272 |
| rs62191444 | T | G | -0.112 | 0.024 | 2.68E-06 | 21.96 | 0.044 | 0.544 |
| rs6756793 | T | C | 0.088 | 0.016 | 2.68E-08 | 31.12 | 0.032 | 0.223 |
| rs6910518 | T | G | 0.081 | 0.016 | 6.01E-07 | 24.75 | 0.032 | 0.855 |
| rs72972467 | C | G | -0.162 | 0.033 | 8.29E-07 | 24.27 | 0.063 | 0.117 |
| rs73162807 | A | C | -0.231 | 0.050 | 3.55E-06 | 21.48 | 0.101 | 0.373 |
| rs9924851 | C | G | 0.077 | 0.016 | 2.70E-06 | 22.14 | 0.033 | 0.923 |
| **RANTES** |  |  |  |  |  |  |  |  |
| rs10505135 | T | C | 0.132 | 0.025 | 1.90E-07 | 27.21 | 0.033 | 0.405 |
| rs118096511 | T | C | -0.337 | 0.071 | 1.97E-06 | 22.63 | 0.094 | 0.656 |
| rs11873385 | A | G | -0.258 | 0.055 | 2.93E-06 | 21.87 | 0.069 | 0.965 |
| rs148526102 | T | C | -0.380 | 0.083 | 4.79E-06 | 20.93 | 0.107 | 0.033 |
| rs2731672 | T | C | -0.124 | 0.027 | 4.83E-06 | 20.84 | 0.036 | 0.721 |
| rs4795087 | C | G | 0.149 | 0.031 | 1.63E-06 | 22.92 | 0.041 | 0.536 |
| rs62438851 | A | G | -0.190 | 0.041 | 4.01E-06 | 21.24 | 0.052 | 0.661 |
| rs7170339 | C | G | -0.428 | 0.090 | 2.19E-06 | 22.43 | 0.112 | 0.270 |
| rs72793342 | A | G | -0.151 | 0.031 | 9.08E-07 | 24.02 | 0.039 | 0.820 |
| rs78050316 | A | C | 0.420 | 0.086 | 9.95E-07 | 23.92 | 0.121 | 0.022 |
| **SCF** |  |  |  |  |  |  |  |  |
| rs10800449 | A | C | 0.085 | 0.018 | 1.96E-06 | 22.60 | 0.035 | 0.249 |
| rs11244035 | T | C | -0.130 | 0.028 | 3.50E-06 | 21.57 | 0.056 | 0.133 |
| rs113127926 | A | C | 0.197 | 0.042 | 2.34E-06 | 22.30 | 0.081 | 0.655 |
| rs117721699 | C | G | -0.239 | 0.048 | 7.51E-07 | 24.42 | 0.099 | 0.628 |
| rs12345108 | T | C | -0.077 | 0.017 | 3.73E-06 | 21.36 | 0.033 | 0.431 |
| rs13412535 | A | G | -0.107 | 0.021 | 5.59E-07 | 24.99 | 0.040 | 0.016 |
| rs138538809 | T | C | -0.579 | 0.114 | 3.76E-07 | 25.82 | 0.218 | 0.694 |
| rs72678285 | A | T | 0.106 | 0.023 | 4.43E-06 | 21.13 | 0.043 | 0.031 |
| rs78666213 | T | G | -0.285 | 0.057 | 7.15E-07 | 24.56 | 0.112 | 0.232 |
| rs8045376 | A | G | -0.313 | 0.068 | 4.27E-06 | 21.13 | 0.140 | 0.825 |
| **SCGFb** |  |  |  |  |  |  |  |  |
| rs11111869 | A | G | 0.162 | 0.031 | 1.86E-07 | 27.15 | 0.042 | 0.819 |
| rs112346514 | T | C | -0.326 | 0.070 | 3.54E-06 | 21.51 | 0.082 | 0.580 |
| rs1149926 | T | C | -0.346 | 0.075 | 3.92E-06 | 21.30 | 0.100 | 0.141 |
| rs118003677 | T | C | -0.365 | 0.079 | 3.35E-06 | 21.60 | 0.103 | 0.065 |
| rs12118918 | A | G | -0.163 | 0.035 | 3.21E-06 | 21.70 | 0.045 | 0.138 |
| rs12480722 | T | C | 0.165 | 0.035 | 2.81E-06 | 21.94 | 0.047 | 0.830 |
| rs13287050 | A | T | -0.121 | 0.026 | 4.12E-06 | 21.16 | 0.035 | 0.939 |
| rs13866 | T | C | -0.165 | 0.028 | 3.77E-09 | 34.58 | 0.036 | 0.759 |
| rs139413256 | A | G | -0.517 | 0.108 | 1.53E-06 | 23.11 | 0.127 | 0.967 |
| rs143829871 | T | C | -0.187 | 0.040 | 2.85E-06 | 21.86 | 0.054 | 0.318 |
| rs144724875 | T | C | 0.538 | 0.083 | 8.65E-11 | 42.11 | 0.095 | 0.232 |
| rs149009264 | A | G | 0.455 | 0.099 | 3.79E-06 | 21.34 | 0.130 | 0.122 |
| rs150733161 | T | C | -0.526 | 0.112 | 2.69E-06 | 22.00 | 0.133 | 0.481 |
| rs151194174 | A | G | 0.454 | 0.094 | 1.45E-06 | 23.22 | 0.081 | 0.344 |
| rs264157 | A | G | 0.108 | 0.023 | 3.69E-06 | 21.43 | 0.031 | 0.658 |
| rs34911860 | A | G | -0.367 | 0.079 | 3.00E-06 | 21.78 | 0.117 | 0.749 |
| rs3817303 | T | G | 0.136 | 0.029 | 3.60E-06 | 21.45 | 0.040 | 0.398 |
| rs4737731 | T | C | 0.115 | 0.025 | 4.87E-06 | 20.83 | 0.035 | 0.658 |
| rs4976691 | C | G | -0.148 | 0.025 | 4.44E-09 | 34.39 | 0.034 | 0.878 |
| rs77954165 | T | C | 0.263 | 0.056 | 2.87E-06 | 21.90 | 0.074 | 0.180 |
| rs78217154 | T | C | 0.394 | 0.086 | 4.72E-06 | 20.95 | 0.113 | 0.984 |
| **SDF1a** |  |  |  |  |  |  |  |  |
| rs10474392 | A | G | 0.093 | 0.018 | 1.38E-07 | 27.84 | 0.036 | 0.400 |
| rs10516368 | A | C | -0.427 | 0.088 | 1.36E-06 | 23.36 | 0.174 | 0.035 |
| rs12141941 | T | C | -0.088 | 0.019 | 2.26E-06 | 22.43 | 0.035 | 0.916 |
| rs149893336 | A | G | -0.494 | 0.108 | 4.93E-06 | 20.84 | 0.151 | 0.407 |
| rs1600396 | A | G | -0.093 | 0.020 | 4.94E-06 | 20.91 | 0.040 | 0.536 |
| rs62194946 | T | G | -0.085 | 0.019 | 4.55E-06 | 21.06 | 0.036 | 0.619 |
| rs6586903 | T | C | -0.126 | 0.027 | 2.42E-06 | 22.24 | 0.051 | 0.089 |
| rs76766406 | A | G | 0.464 | 0.101 | 4.49E-06 | 21.03 | 0.193 | 0.687 |
| rs78883416 | C | G | -0.087 | 0.018 | 1.76E-06 | 22.90 | 0.034 | 0.692 |
| **TNFa** |  |  |  |  |  |  |  |  |
| rs10767536 | A | G | 0.118 | 0.033 | 3.15E-06 | 21.74 | 0.025 | 0.045 |
| rs115018697 | C | G | -0.954 | 0.249 | 1.27E-06 | 23.45 | 0.197 | 0.576 |
| rs116736594 | T | C | 0.341 | 0.086 | 1.22E-06 | 23.54 | 0.070 | 0.472 |
| rs79105320 | A | G | 0.557 | 0.150 | 2.21E-06 | 22.41 | 0.118 | 0.939 |
| **TNFb** |  |  |  |  |  |  |  |  |
| rs10925040 | T | C | 0.174 | 0.037 | 2.93E-06 | 21.80 | 0.033 | 0.473 |
| rs2420873 | T | G | 0.167 | 0.037 | 4.51E-06 | 20.98 | 0.032 | 0.617 |
| rs62284710 | A | G | 0.370 | 0.078 | 2.18E-06 | 22.38 | 0.066 | 0.262 |
| rs75240021 | C | G | 0.371 | 0.077 | 1.49E-06 | 23.10 | 0.059 | 0.315 |
| rs76225863 | A | G | 0.753 | 0.122 | 5.98E-10 | 38.27 | 0.099 | 0.745 |
| **TRAIL** |  |  |  |  |  |  |  |  |
| rs113057689 | A | G | -0.263 | 0.049 | 7.97E-08 | 28.81 | 0.076 | 0.454 |
| rs11875481 | T | C | -0.097 | 0.021 | 4.60E-06 | 21.09 | 0.042 | 0.336 |
| rs12458564 | A | T | -0.100 | 0.018 | 1.10E-08 | 32.78 | 0.035 | 0.317 |
| rs13115587 | A | C | 0.101 | 0.022 | 3.24E-06 | 21.66 | 0.044 | 0.969 |
| rs13278062 | T | G | 0.080 | 0.016 | 3.33E-07 | 25.96 | 0.032 | 0.948 |
| rs139958028 | A | G | 0.180 | 0.040 | 4.99E-06 | 20.83 | 0.076 | 0.515 |
| rs183815186 | A | T | -0.350 | 0.060 | 6.34E-09 | 33.77 | 0.116 | 0.360 |
| rs550057 | T | C | -0.078 | 0.017 | 3.71E-06 | 21.46 | 0.034 | 0.702 |
| rs558572 | T | C | 0.135 | 0.027 | 3.42E-07 | 25.98 | 0.053 | 0.040 |
| rs57396456 | T | C | -0.564 | 0.052 | 7.71E-28 | 119.48 | 0.103 | 0.726 |
| rs616114 | T | C | -0.103 | 0.016 | 1.72E-10 | 40.65 | 0.032 | 0.676 |
| rs62093482 | T | C | 0.983 | 0.053 | 6.12E-77 | 345.00 | 0.109 | 0.183 |
| rs747324 | T | C | -0.083 | 0.018 | 3.34E-06 | 21.53 | 0.035 | 0.082 |
| rs75928541 | A | G | 0.278 | 0.059 | 2.44E-06 | 22.18 | 0.107 | 0.588 |
| rs7599203 | T | C | 0.092 | 0.020 | 4.33E-06 | 21.06 | 0.040 | 0.414 |
| rs78682108 | A | G | -0.238 | 0.039 | 1.44E-09 | 36.57 | 0.073 | 0.626 |
| **VEGF** |  |  |  |  |  |  |  |  |
| rs10411345 | C | G | -0.104 | 0.022 | 1.73E-06 | 22.80 | 0.037 | 0.802 |
| rs10757514 | C | G | -0.102 | 0.022 | 4.17E-06 | 21.27 | 0.041 | 0.332 |
| rs10822118 | T | C | -0.080 | 0.017 | 2.21E-06 | 22.50 | 0.031 | 0.486 |
| rs10934631 | T | C | -0.113 | 0.024 | 3.61E-06 | 21.52 | 0.044 | 0.404 |
| rs114773511 | T | C | 0.219 | 0.044 | 6.97E-07 | 24.59 | 0.077 | 0.801 |
| rs12456390 | T | C | -0.082 | 0.018 | 4.88E-06 | 20.88 | 0.034 | 0.061 |
| rs1730969 | C | G | -0.781 | 0.170 | 4.11E-06 | 21.20 | 0.381 | 0.290 |
| rs181031888 | A | T | 0.377 | 0.050 | 4.01E-14 | 57.20 | 0.086 | 0.912 |
| rs2039420 | C | G | 0.098 | 0.018 | 2.74E-08 | 31.03 | 0.033 | 0.293 |
| rs56071907 | T | C | 0.126 | 0.027 | 3.01E-06 | 21.77 | 0.045 | 0.692 |
| rs60013354 | A | G | -0.250 | 0.052 | 1.66E-06 | 22.96 | 0.095 | 0.399 |
| rs62401205 | A | C | -0.202 | 0.041 | 9.95E-07 | 23.91 | 0.063 | 0.940 |
| rs6496613 | A | C | -0.236 | 0.052 | 4.70E-06 | 20.98 | 0.097 | 0.474 |
| rs7356919 | A | G | -0.169 | 0.020 | 2.85E-17 | 71.72 | 0.037 | 0.157 |
| rs73872715 | T | C | -0.608 | 0.130 | 2.86E-06 | 21.89 | 0.211 | 0.169 |
| rs76458389 | T | G | -0.179 | 0.036 | 8.57E-07 | 24.20 | 0.066 | 0.854 |
| rs77961527 | A | G | 0.229 | 0.046 | 5.53E-07 | 25.08 | 0.074 | 0.867 |
| rs9381249 | T | C | -0.241 | 0.040 | 1.04E-09 | 37.15 | 0.079 | 0.748 |

# Table S4. MR estimates of PD on forty-one inflammatory cytokines.

| **Inverse variance weighted** | | | | | | **MR-Egger** | | | **Weighted Median** | | | **Simple Mode** | | | **Weighted Mode** | | |
| --- | --- | --- | --- | --- | --- | --- | --- | --- | --- | --- | --- | --- | --- | --- | --- | --- | --- |
| **Category** | **Outcomes** | **SNPs** | **OR** | **95% CI** | **pval** | **OR** | **95% CI** | **pval** | **OR** | **95% CI** | **pval** | **OR** | **95% CI** | **pval** | **OR** | **95% CI** | **pval** |
| **Chemokines** | | | | | | | | | | | | | | | | | |
|  | CTACK | 21 | 1.010 | (0.94-1.09) | 0.783 | 1.036 | (0.89-1.21) | 0.654 | 1.015 | (0.91-1.13) | 0.774 | 1.064 | (0.90-1.25) | 0.460 | 1.028 | 1(0.91-1.16) | 0.659 |
|  | Eotaxin | 21 | 0.963 | (0.92-1.01) | 0.129 | 0.981 | (0.89-1.09) | 0.720 | 0.974 | (0.90-1.05) | 0.491 | 0.979 | (0.86-1.11) | 0.740 | 0.977 | (0.90-1.07) | 0.606 |
|  | GROa | 21 | 1.006 | (0.93-1.08) | 0.878 | 1.009 | (0.86-1.18) | 0.910 | 1.011 | (0.91-1.12) | 0.838 | 1.065 | (0.90-1.26) | 0.468 | 1.019 | (0.91-1.14) | 0.745 |
|  | IP10 | 21 | 0.983 | (0.91-1.06) | 0.649 | 0.948 | (0.81-1.10) | 0.503 | 1.011 | (0.91-1.12) | 0.838 | 1.057 | (0.89-1.25) | 0.529 | 1.007 | (0.89-1.14) | 0.922 |
|  | MCP1 | 21 | 0.998 | (0.95-1.05) | 0.949 | 0.991 | (0.89-1.10) | 0.868 | 1.011 | (0.94-1.08) | 0.761 | 1.041 | (0.91-1.19) | 0.572 | 1.000 | (0.92-1.09) | 0.993 |
|  | MCP3 | 21 | 1.053 | (0.91-1.22) | 0.474 | 0.892 | (0.66-1.21) | 0.473 | 1.021 | (0.84-1.25) | 0.836 | 1.073 | (0.76-1.51) | 0.691 | 1.023 | (0.82-1.28) | 0.844 |
|  | MIG | 21 | 0.910 | (0.84-0.98) | **0.014** | 0.907 | (0.77-1.07) | 0.252 | 0.903 | (0.81-1.01) | 0.062 | 0.855 | (0.69-1.05) | 0.158 | 0.904 | (0.80-1.02) | 0.116 |
|  | MIP1a | 21 | 0.963 | (0.89-1.04) | 0.322 | 0.991 | (0.85-1.16) | 0.912 | 0.968 | (0.87-1.07) | 0.536 | 0.967 | (0.80-1.17) | 0.735 | 0.994 | (0.86-1.14) | 0.938 |
|  | MIP1b | 21 | 0.975 | (0.93-1.02) | 0.302 | 0.961 | (0.87-1.06) | 0.452 | 0.983 | (0.92-1.06) | 0.639 | 0.885 | (0.78-1.00) | 0.066 | 0.981 | (0.91-1.05) | 0.604 |
|  | RANTES | 21 | 1.038 | (0.96-1.12) | 0.337 | 0.923 | (0.79-1.08) | 0.334 | 0.980 | (0.88-1.09) | 0.726 | 0.926 | (0.75-1.14) | 0.477 | 0.953 | (0.84-1.09) | 0.479 |
|  | SDF1a | 21 | 0.973 | (0.93-1.02) | 0.288 | 1.030 | (0.93-1.14) | 0.584 | 0.996 | (0.93-1.07) | 0.916 | 0.892 | (0.77-1.03) | 0.128 | 1.008 | (0.92-1.10) | 0.860 |
| **Growth factors** | | | | | | | | | | | | | | | | | |
|  | bNGF | 21 | 0.915 | (0.85-0.99) | **0.019** | 1.021 | (0.87-1.19) | 0.795 | 0.967 | (0.87-1.07) | 0.527 | 0.885 | (0.74-1.06) | 0.209 | 0.972 | (0.87-1.09) | 0.634 |
|  | FGFBasic | 21 | 0.966 | (0.92-1.02) | 0.202 | 0.978 | (0.87-1.10) | 0.711 | 1.009 | (0.93-1.09) | 0.821 | 0.985 | (0.87-1.11) | 0.814 | 1.008 | (0.93-1.09) | 0.846 |
|  | GCSF | 21 | 1.008 | (0.96-1.06) | 0.739 | 0.997 | (0.90-1.11) | 0.953 | 1.020 | (0.95-1.09) | 0.582 | 0.984 | (0.86-1.12) | 0.806 | 1.020 | (0.94-1.10) | 0.624 |
|  | HGF | 21 | 0.962 | (0.91-1.02) | 0.172 | 0.920 | (0.82-1.03) | 0.181 | 0.970 | (0.90-1.04) | 0.401 | 1.021 | (0.91-1.15) | 0.733 | 0.963 | (0.89-1.04) | 0.346 |
|  | MCSF | 21 | 0.987 | (0.89-1.10) | 0.814 | 1.022 | (0.81-1.29) | 0.852 | 1.037 | (0.91-1.18) | 0.573 | 1.006 | (0.78-1.30) | 0.966 | 1.022 | (0.88-1.19) | 0.777 |
|  | PDGFbb | 21 | 0.988 | (0.94-1.04) | 0.614 | 0.990 | (0.89-1.09) | 0.843 | 0.996 | (0.93-1.07) | 0.901 | 0.987 | (0.87-1.12) | 0.846 | 1.004 | (0.93-1.09) | 0.918 |
|  | SCF | 21 | 0.953 | (0.91-1.00) | 0.065 | 0.914 | (0.82-1.02) | 0.114 | 0.922 | (0.86-0.99) | 0.023 | 0.905 | (0.80-1.02) | 0.122 | 0.917 | (0.85-0.99) | 0.029 |
|  | SCGFb | 21 | 0.975 | (0.89-1.06) | 0.571 | 1.005 | (0.84-1.21) | 0.956 | 1.023 | (0.92-1.14) | 0.675 | 1.064 | (0.92-1.23) | 0.419 | 1.039 | (0.92-1.17) | 0.524 |
|  | VEGF | 21 | 0.989 | (0.93-1.05) | 0.704 | 0.938 | (0.83-1.06) | 0.314 | 0.997 | (0.93-1.07) | 0.933 | 1.005 | (0.90-1.13) | 0.928 | 0.999 | (0.92-1.08) | 0.980 |
| **Interleukins** | | | | | | | | | | | | | | | | | |
|  | IL10 | 21 | 0.978 | (0.93-1.03) | 0.426 | 0.930 | (0.83-1.04) | 0.219 | 0.965 | (0.90-1.04) | 0.328 | 0.967 | (0.87-1.07) | 0.540 | 0.969 | (0.90-1.04) | 0.412 |
|  | IL12p70 | 21 | 0.974 | (0.93-1.03) | 0.313 | 0.980 | (0.88-1.09) | 0.720 | 0.985 | (0.91-1.06) | 0.701 | 0.911 | (0.79-1.04) | 0.195 | 0.985 | (0.91-1.07) | 0.719 |
|  | IL13 | 21 | 0.984 | (0.91-1.06) | 0.672 | 1.011 | (0.87-1.18) | 0.892 | 1.001 | (0.90-1.12) | 0.981 | 0.988 | (0.82-1.19) | 0.904 | 1.001 | (0.88-1.14) | 0.987 |
|  | IL16 | 21 | 0.976 | (0.91-1.05) | 0.522 | 1.140 | (0.98-1.33) | 0.116 | 1.046 | (0.94-1.16) | 0.402 | 1.011 | (0.83-1.24) | 0.919 | 1.059 | (0.94-1.19) | 0.341 |
|  | IL17 | 21 | 0.939 | (0.89-0.99) | **0.028** | 0.930 | (0.83-1.05) | 0.242 | 0.954 | (0.88-1.03) | 0.228 | 0.863 | (0.76-0.98) | 0.038 | 0.959 | (0.89-1.03) | 0.292 |
|  | IL18 | 21 | 0.970 | (0.89-1.06) | 0.475 | 1.005 | (0.84-1.20) | 0.961 | 0.966 | (0.87-1.08) | 0.531 | 1.021 | (0.85-1.22) | 0.823 | 0.976 | (0.87-1.10) | 0.698 |
|  | IL1b | 21 | 0.966 | (0.89-1.04) | 0.371 | 0.927 | (0.79-1.09) | 0.368 | 0.947 | (0.85-1.06) | 0.327 | 0.948 | (0.80-1.12) | 0.537 | 0.938 | (0.81-1.08) | 0.381 |
|  | IL1ra | 21 | 0.950 | (0.88-1.02) | 0.174 | 0.972 | (0.83-1.13) | 0.725 | 0.964 | (0.87-1.07) | 0.500 | 0.929 | (0.78-1.11) | 0.434 | 0.972 | (0.87-1.09) | 0.636 |
|  | IL2 | 21 | 0.923 | (0.86-0.99) | 0.036 | 0.951 | (0.81-1.11) | 0.537 | 0.950 | (0.85-1.06) | 0.342 | 1.002 | (0.84-1.20) | 0.979 | 0.972 | (0.86-1.09) | 0.646 |
|  | IL2ra | 21 | 1.009 | (0.94-1.09) | 0.808 | 1.123 | (0.96-1.31) | 0.151 | 0.981 | (0.88-1.09) | 0.721 | 0.900 | (0.72-1.12) | 0.359 | 0.933 | (0.78-1.11) | 0.444 |
|  | IL4 | 21 | 0.987 | (0.94-1.04) | 0.591 | 0.974 | (0.88-1.08) | 0.615 | 1.013 | (0.94-1.09) | 0.720 | 1.020 | (0.91-1.14) | 0.729 | 1.022 | (0.94-1.11) | 0.627 |
|  | IL5 | 21 | 0.993 | (0.92-1.07) | 0.857 | 1.051 | (0.89-1.24) | 0.570 | 1.074 | (0.96-1.20) | 0.214 | 1.046 | (0.87-1.26) | 0.646 | 1.083 | (0.96-1.22) | 0.205 |
|  | IL6 | 21 | 0.977 | (0.93-1.03) | 0.378 | 0.947 | (0.85-1.05) | 0.331 | 0.966 | (0.90-1.04) | 0.352 | 0.994 | (0.88-1.12) | 0.927 | 0.983 | (0.91-1.06) | 0.658 |
|  | IL7 | 21 | 1.006 | (0.93-1.08) | 0.881 | 0.991 | (0.85-1.16) | 0.910 | 0.960 | (0.86-1.07) | 0.462 | 0.949 | (0.78-1.15) | 0.609 | 0.949 | (0.82-1.10) | 0.496 |
|  | IL8 | 21 | 1.000 | (0.94-1.06） | 0.990 | 1.041 | (0.95-1.13） | 0.518 | 1.006 | (0.96-1.05） | 0.798 | 1.026 | (0.92-1.13） | 0.659 | 1.008 | (0.96-1.051） | 0.750 |
|  | IL9 | 21 | 0.961 | (0.89-1.03) | 0.290 | 0.946 | (0.81-1.10) | 0.488 | 0.946 | (0.86-1.05) | 0.278 | 0.938 | (0.78-1.13) | 0.518 | 0.945 | (0.82-1.08) | 0.423 |
| **Others** | | | | | | | | | | | | | | | | | |
|  | IFNg | 21 | 0.950 | (0.90-1.00) | **0.044** | 0.920 | (0.83-1.02) | 0.130 | 0.949 | (0.88-1.02) | 0.141 | 0.915 | (0.82-1.02) | 0.135 | 0.935 | (0.87-1.01) | 0.097 |
|  | MIF | 21 | 0.996 | (0.92-1.07) | 0.909 | 1.055 | (0.90-1.23) | 0.508 | 0.999 | (0.90-1.11) | 0.981 | 0.945 | (0.78-1.14) | 0.561 | 0.979 | (0.85-1.13) | 0.767 |
|  | TNFa | 21 | 0.987 | (0.92-1.06) | 0.724 | 0.989 | (0.85-1.16) | 0.892 | 0.979 | (0.88-1.10) | 0.709 | 0.880 | (0.72-1.07) | 0.217 | 0.976 | (0.85-1.12) | 0.726 |
|  | TNFb | 21 | 1.031 | (0.92-1.16) | 0.606 | 0.932 | (0.73-1.19) | 0.583 | 0.965 | (0.82-1.14) | 0.677 | 1.101 | (0.86-1.41) | 0.456 | 0.968 | (0.81-1.15) | 0.713 |
|  | TRAIL | 21 | 1.017 | (0.97-1.07) | 0.488 | 1.033 | (0.93-1.14) | 0.541 | 0.995 | (0.93-1.06) | 0.876 | 0.996 | (0.89-1.11) | 0.945 | 1.003 | (0.93-1.08) | 0.941 |

# Table S5. Heterogeneity and horizontal pleiotropy tests of PD on forty-one inflammatory cytokines.

| **Cytokine** | **Q_1_ pval** | **Q_2_ pval** | **intercept** | **intercept pval** | **Recommended Method** |
| --- | --- | --- | --- | --- | --- |
| bNGF | 0.704 | 0.799 | -0.021 | 0.133 | IVW |
| CTACK | 0.695 | 0.643 | -0.005 | 0.716 | IVW |
| Eotaxin | 0.616 | 0.564 | -0.004 | 0.680 | IVW |
| FGFBasic | 0.319 | 0.268 | -0.003 | 0.800 | IVW |
| GCSF | 0.468 | 0.408 | 0.009 | 0.807 | IVW |
| GROa | 0.881 | 0.843 | -0.001 | 0.964 | IVW |
| HGF | 0.139 | 0.133 | 0.009 | 0.412 | IVW |
| IFNg | 0.766 | 0.741 | 0.006 | 0.498 | IVW |
| IL10 | 0.265 | 0.270 | 0.010 | 0.325 | IVW |
| IL12p70 | 0.321 | 0.267 | -0.001 | 0.902 | IVW |
| IL13 | 0.737 | 0.689 | -0.005 | 0.704 | IVW |
| IL16 | 0.921 | 0.995 | -0.030 | 0.038 | IVW |
| IL17 | 0.204 | 0.164 | 0.002 | 0.846 | IVW |
| IL18 | 0.146 | 0.120 | -0.007 | 0.668 | IVW |
| IL1b | 0.932 | 0.918 | 0.008 | 0.581 | IVW |
| IL1ra | 0.880 | 0.848 | -0.004 | 0.744 | IVW |
| IL2 | 0.968 | 0.957 | -0.006 | 0.679 | IVW |
| IL2ra | 0.563 | 0.664 | -0.021 | 0.133 | IVW |
| IL4 | 0.572 | 0.513 | 0.003 | 0.776 | IVW |
| IL5 | 0.364 | 0.340 | -0.011 | 0.460 | IVW |
| IL6 | 0.330 | 0.300 | 0.006 | 0.517 | IVW |
| IL7 | 0.686 | 0.628 | 0.003 | 0.835 | IVW |
| IL8 | 0.24 | 0.18 | -0.041 | 0.437 | IVW |
| IL9 | 0.818 | 0.773 | 0.003 | 0.818 | IVW |
| IP10 | 0.760 | 0.723 | 0.007 | 0.602 | IVW |
| MCP1 | 0.360 | 0.304 | 0.001 | 0.876 | IVW |
| MCP3 | 0.335 | 0.365 | 0.031 | 0.243 | IVW |
| MCSF | 0.079 | 0.061 | -0.007 | 0.738 | IVW |
| MIF | 0.788 | 0.777 | -0.011 | 0.418 | IVW |
| MIG | 0.365 | 0.307 | 0.001 | 0.968 | IVW |
| MIP1a | 0.710 | 0.662 | -0.006 | 0.685 | IVW |
| MIP1b | 0.514 | 0.456 | 0.003 | 0.760 | IVW |
| PDGFbb | 0.811 | 0.761 | -0.0004 | 0.962 | IVW |
| RANTES | 0.567 | 0.684 | 0.023 | 0.116 | IVW |
| SCF | 0.321 | 0.310 | 0.008 | 0.389 | IVW |
| SCGFb | 0.106 | 0.084 | -0.006 | 0.720 | IVW |
| SDF1a | 0.478 | 0.510 | -0.011 | 0.238 | IVW |
| TNFa | 0.511 | 0.446 | -0.0005 | 0.973 | IVW |
| TNFb | 0.700 | 0.694 | 0.019 | 0.380 | IVW |
| TRAIL | 0.821 | 0.779 | -0.003 | 0.746 | IVW |
| VEGF | 0.187 | 0.189 | 0.010 | 0.343 | IVW |

# Table S6. Detail information of instrumental variables of PD

| **SNP** | **effect allele** | **other allele** | **Chrom** | **EAF** | **F** | **pval** | **se** |
| --- | --- | --- | --- | --- | --- | --- | --- |
| rs10513789 | G | T | 3 | 0.1826 | 53.11 | 3.18E-13 | 0.0219 |
| rs10847864 | T | G | 12 | 0.3625 | 50.66 | 9.81E-13 | 0.0179 |
| rs12934900 | T | A | 16 | 0.6571 | 43.60 | 4.33E-11 | 0.0184 |
| rs144814361 | T | C | 10 | 0.0174 | 42.08 | 9.07E-11 | 0.068 |
| rs329647 | C | G | 11 | 0.6662 | 40.52 | 1.94E-10 | 0.0178 |
| rs34311866 | C | T | 4 | 0.1958 | 96.74 | 7.97E-23 | 0.0231 |
| rs35265698 | G | C | 6 | 0.1547 | 43.57 | 3.93E-11 | 0.0303 |
| rs356203 | T | C | 4 | 0.6169 | 181.49 | 3.01E-41 | 0.0178 |
| rs35749011 | A | G | 1 | 0.0191 | 129.80 | 5.02E-30 | 0.0659 |
| rs4488803 | A | G | 3 | 0.3746 | 32.59 | 1.08E-08 | 0.0199 |
| rs4588066 | A | G | 18 | 0.326 | 34.53 | 4.45E-09 | 0.0178 |
| rs4613239 | G | C | 2 | 0.1326 | 51.75 | 6.21E-13 | 0.0248 |
| rs4698412 | A | G | 4 | 0.553 | 56.07 | 7.05E-14 | 0.0168 |
| rs4774417 | A | G | 15 | 0.7397 | 30.02 | 4.63E-08 | 0.0192 |
| rs620490 | G | T | 8 | 0.2762 | 38.18 | 6.46E-10 | 0.019 |
| rs6741007 | G | T | 2 | 0.4507 | 49.64 | 2.09E-12 | 0.0175 |
| rs75505347 | T | C | 12 | 0.0195 | 33.77 | 6.12E-09 | 0.0674 |
| rs75646569 | G | T | 5 | 0.1117 | 51.88 | 5.62E-13 | 0.0266 |
| rs7695720 | C | A | 4 | 0.2091 | 36.40 | 1.53E-09 | 0.0208 |
| rs823106 | C | G | 1 | 0.8488 | 38.97 | 4.10E-10 | 0.0239 |
| rs858295 | G | A | 7 | 0.3947 | 34.85 | 3.83E-09 | 0.0176 |


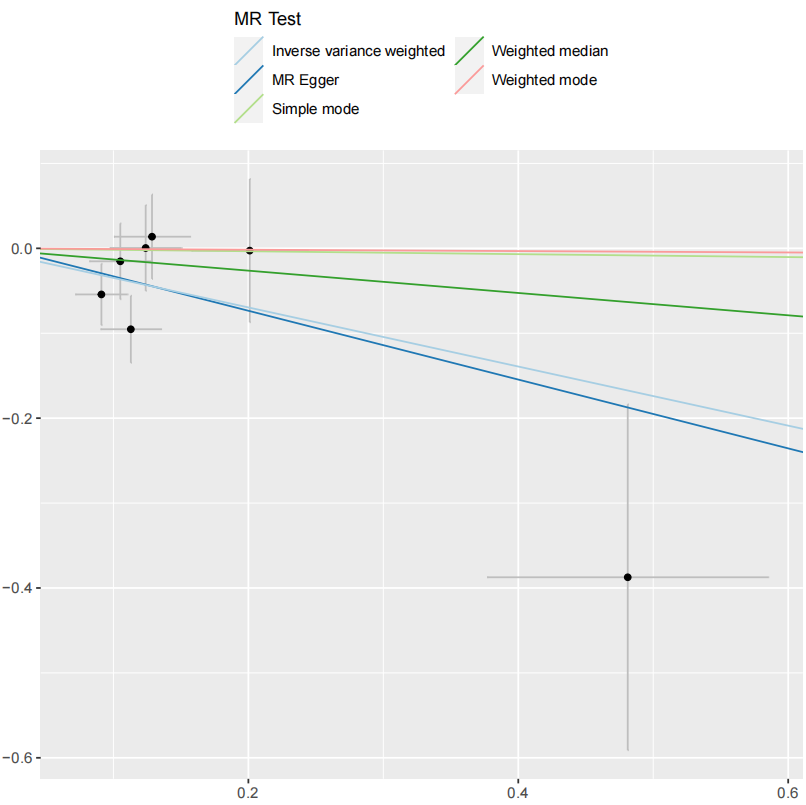


**Figure S1** Scatter plots of Mendelian randomization analyses for FGFBasic on PD.


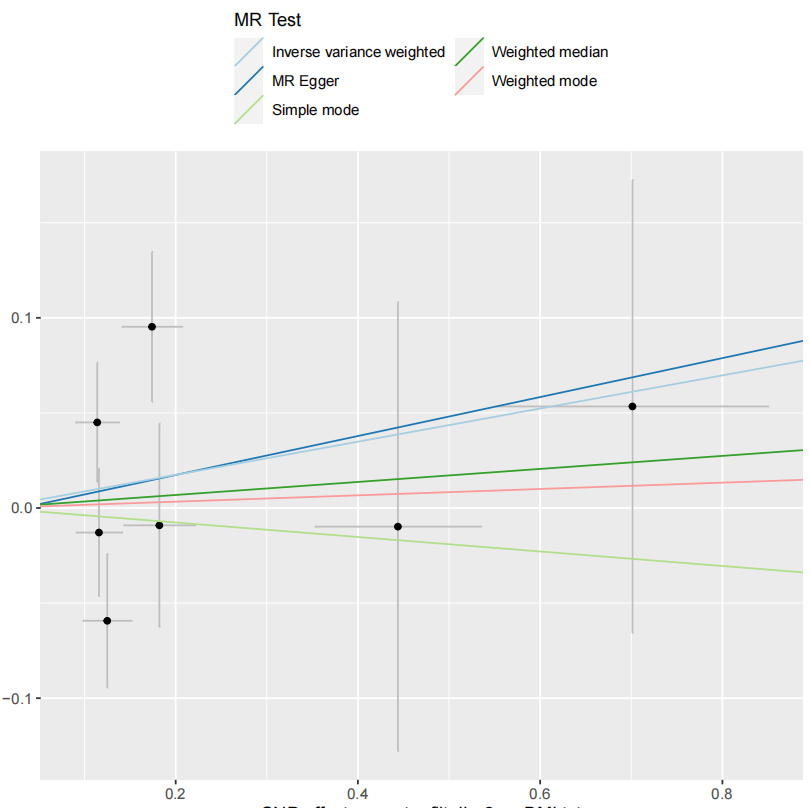


**Figure S2** Scatter plots of Mendelian randomization analyses for IL-2 on PD.


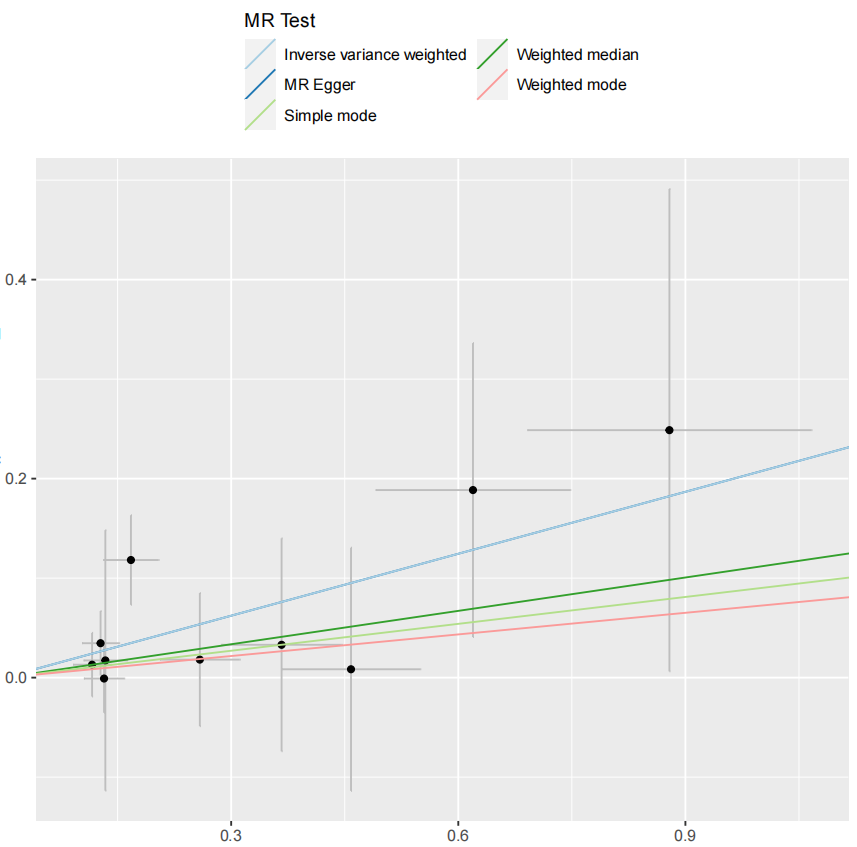


**Figure S3** Scatter plots of Mendelian randomization analyses for MIF on PD.


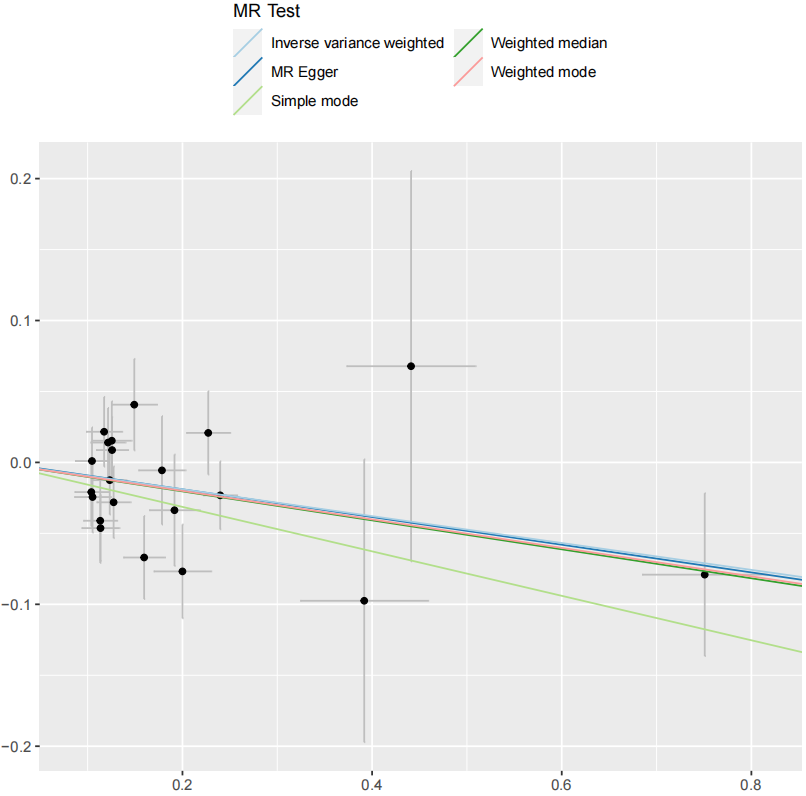


**Figure S4** Scatter plots of Mendelian randomization analyses for PD on MIG.


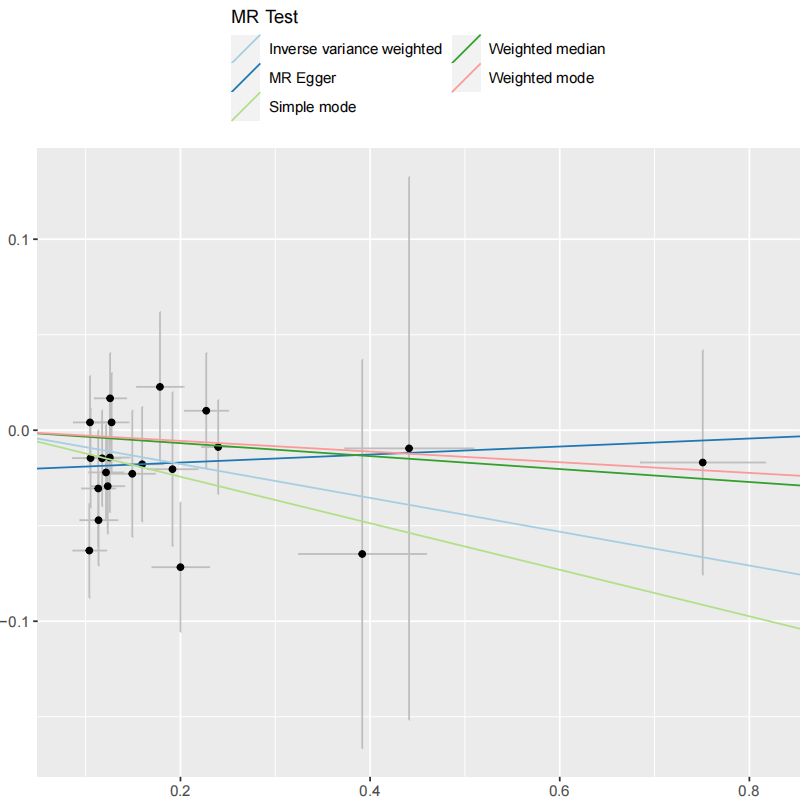


**Figure S5** Scatter plots of Mendelian randomization analyses for PD on bNGF.


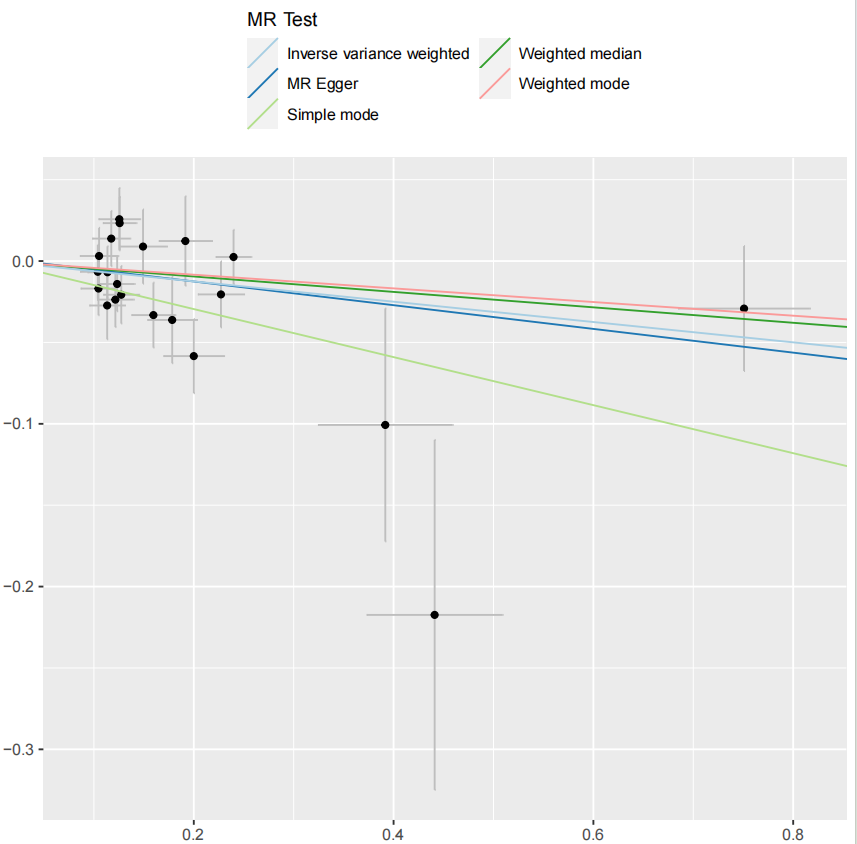


**Figure S6** Scatter plots of Mendelian randomization analyses for PD on IL-17.


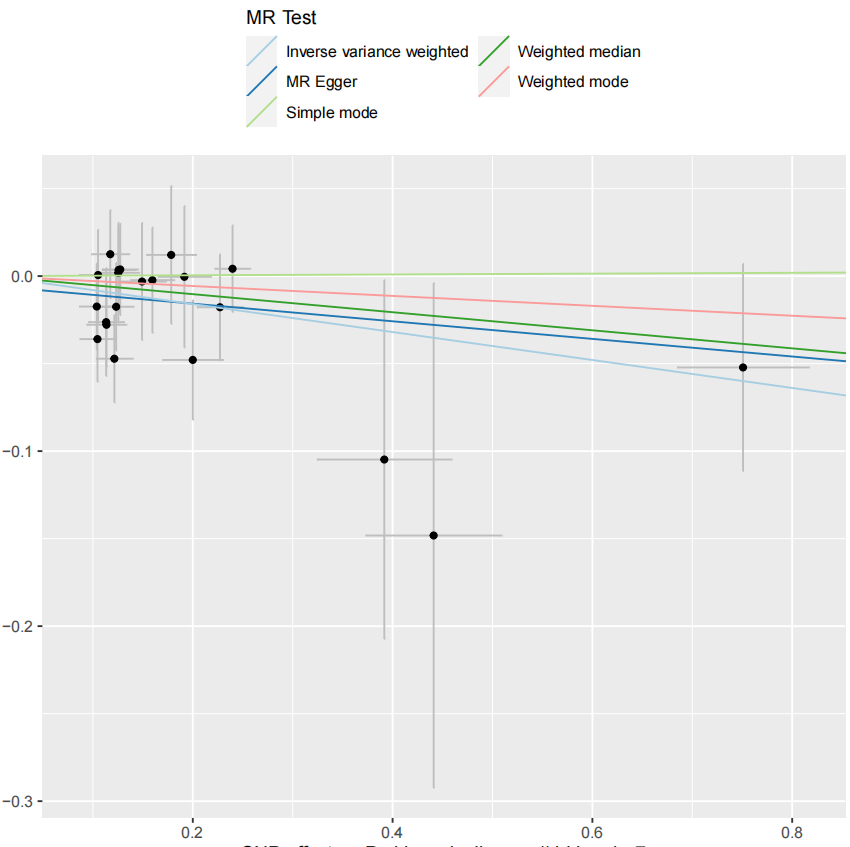


**Figure S7** Scatter plots of Mendelian randomization analyses for PD on IL-2.


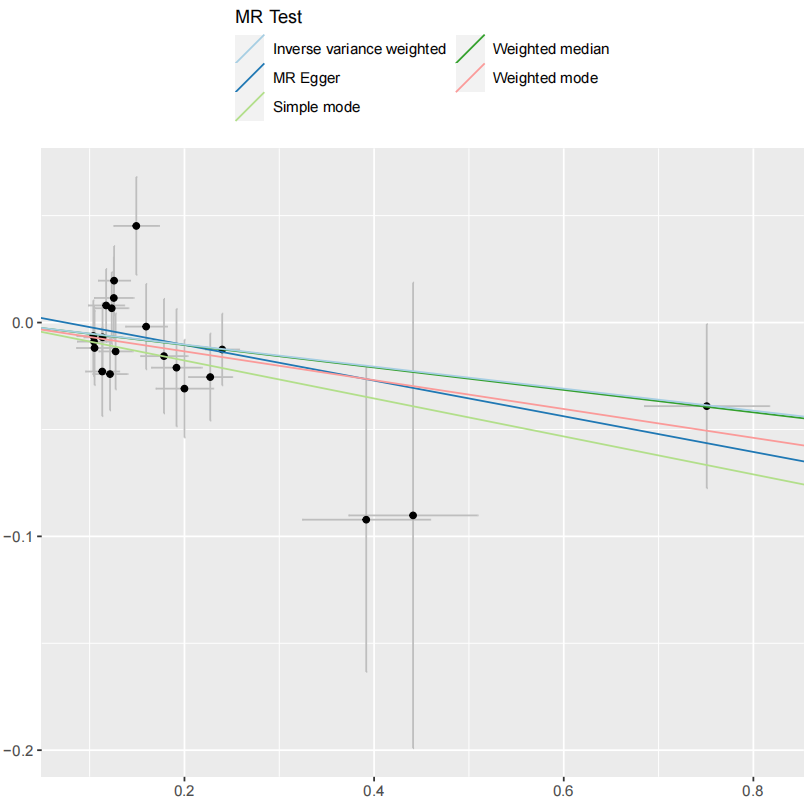


**Figure S8** Scatter plots of Mendelian randomization analyses for PD on IFNg.
